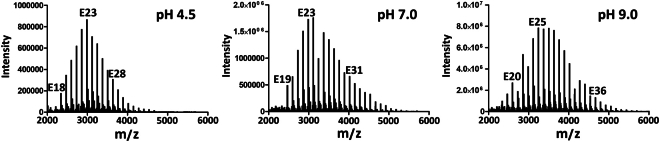# A retropepsin-like bacterial protease regulates ribosome modification and polypeptide production

**DOI:** 10.1016/j.jbc.2025.108329

**Published:** 2025-02-18

**Authors:** Richard H. Little, Govind Chandra, Gerhard Saalbach, Carlo Martins, Catriona M.A. Thompson, Jacob G. Malone

**Affiliations:** 1 Department of Molecular Microbiology, John Innes Centre, Norwich, UK; 2 School of Biological Sciences, University of East Anglia, Norwich, UK

**Keywords:** aspartic peptidase, ibosomal modification, poly-α-L-glutamate, *Pseudomonas*, retropepsin protease

## Abstract

Adaptations to fluctuating environmental conditions require bacteria to make large-scale proteomic shifts on short timescales. We previously characterised the tri-partite RimABK protein complex responsible for the post-translational modification of the ribosome in response to environmental cues. Regulated control of RpsF polyglutamylation by RimK rapidly influenced the proteome of *Pseudomonas fluorescens* cells to facilitate colonisation of the plant rhizosphere. Here, we conduct a detailed investigation of the RimB protease. We show RimB to be a bifunctional retropepsin-like aspartic endopeptidase that uniquely recognises and removes glutamate residues from polyglutamated RpsF and stimulates poly-α-L-glutamate synthesis by RimK. We determine the minimal recognition requirements for RimB proteolysis and identify the catalytic aspartate residue required for function. Further, we identify a novel hybrid enzyme composed of RimB and RimK domains that also possesses protease activity. Phylogenetic analysis of accessions encoding either the hybrid or individual RimB and RimK proteins reveals a pattern of *rim* gene evolution that is distinct from that of the host organisms and reveals potential alternative targets of RimB.

Bacterial proteases are ubiquitous and diverse enzymes capable of performing highly specialized processing of specific substrates. Protease activity controls every aspect of cellular function, creates new bioactive molecules, and serves critical virulence functions (1). Approximately 3% of the *Pseudomonas aeruginosa* genome is composed of open reading frames encoding proteolytic enzymes (2, 3). Thus, proteases are obvious, abundant, but relatively underexplored antimicrobial targets, with potential applications to synthetic biology.

Aspartic peptidases are protease enzymes belonging to the AA clade of proteases that utilize a catalytic dyad of aspartate residues and a water molecule to hydrolyze peptide bonds (4). This clade is unknown in all but a few prokaryotes and may have arisen in an early eukaryote (5, 6). Pepsin-like *MEROPS* family A1 aspartic endopeptidases have low primary sequence similarity and are characterized by two lobes with similar folds, each containing a motif Xaa-Xaa-Asp-Xbb-Gly-Xbb where Xaa is a hydrophobic amino acid and Xbb is either Ser. or Thr. Each lobe contributes one catalytic aspartate residue (7).

Family A2 includes the peptidase from the human immunodeficiency virus (retropepsin, EC 3.4.23.16) and other retroviruses. Retroviral and retrotransposon proteases are homodimers and homologous to a single domain of the pepsin-like proteases. Each domain contributes a catalytic Asp residue, with an extended active site cleft localized between the two lobes of the molecule (8). Optimal catalysis occurs at a higher pH than for typical aspartic proteases (9). Those rare examples of known and characterized bacterial A2 family peptidases reveal an involvement of other protein factors to activate protease activity or to participate in a catabolic cascade (10).

Proteolytic complexes have been identified whereby a protease associates with an activating partner to catabolize cellular targets. Such examples include carboxy-terminal targeting serine protease (CTP) CtpA of *P. aeruginosa*, which forms a complex with LbcA to target cell wall hydrolases (11). The AAA + family proteolytic complexes comprise a serine protease organized into an inactive stacked ring conformation and a specificity determinant belonging to the AAA + superfamily of proteins, which tightly regulates conformational activation of the protease in an ATP-dependent manner, typified by the Clp proteolytic complex (12).

Examples of protein complexes acting as modules for the dynamic regulation of a target by reversible addition of amino acids are rarer in biology. In such systems, the post-translational addition of side-chain amino acids is balanced by the activity of a cognate protease. A paradigm for this behavior is exemplified by the tailored modification of tubulin managed by the opposing activities of γ/α-glutamylases and deglutamylases, including those belonging to the CTP family of proteases (13, 14).

Interestingly, polyglutamylation is emerging as a potential fine regulator of diverse cellular processes in eukaryotes (15) where the utility of this modification is hypothesized to control the affinity of targets for their binding partners (16). The identification of this phenomenon in prokaryotes however remains at a nascent stage. A recent study identified the side chain addition of glutamate residues to SdeA of *Legionella pneumophila* by the pseudokinase SidJ to prevent ADP-ribosylation activity (17). Uniquely, observance of C-terminal protein extension by poly-α-L-glutamylation is thus far limited to the post-translational modification of the ribosomal binding protein S6 (hereafter called RpsF) (18, 19, 20).

RpsF resides in the small (30S) subunit of the bacterial ribosome responsible for binding and decoding mRNA during translation (21). In addition to interacting with RNA helix 23 of the central domain, RpsF binds to ribosomal protein S18 (22, 23). The mobile tails of RpsF and S18 interact with the weakly associating ribosomal protein S1 that interacts with the mRNA entrance and exit channels (24). Furthermore, protein S1 has been demonstrated to interact with RNA polymerase and is an integral part of the Q phage replisome in *Escherichia coli* (3, 25, 26). In both cases, the global post-transcriptional regulator of metabolism, Hfq was shown to be an integral component of the protein complexes (25, 27).

Glutamylation of RpsF was first described in *E. coli* where the activity of a monocistronic ATP-Grasp superfamily member, RimK was demonstrated to add a small number of glutamate residues (numbering around 4) to the C-terminus of RpsF although the purpose of this modification was unknown (28). Furthermore, RimK was shown to catalyze the polymerization of L-glutamate *in vitro* (29). We recently described the environmentally regulated *rimABK* operon of *Pseudomonas fluorescens* SBW25. RimK activity directly influences translation through ribosomal modification and alters Hfq levels in a media-dependent manner. Both activities result in the remodeling of the proteome to a state that is favored by the environmental conditions encountered by the cell (19, 20, 30, 31).

*rimK* is co-transcribed with *rimA* encoding a phosphodiesterase enzyme RimA, active in hydrolyzing the second messenger cyclic-di-GMP (cdG), and *rimB* encoding a protease enzyme, RimB. The binding of cdG to RimK together with stoichiometric interaction between RimK and RimA additively stimulates RpsF modification. However, increasing concentrations of cdG diminish RimA stimulation of RimK activity. Thus, glutamylation of a ribosome occurs in a cdG-dependent manner with RimA acting as a trigger enzyme by reducing stimulation of RimK as its ability to hydrolyze cdG is outpaced by rising concentrations of the second messenger. To prevent unlimited processive addition of glutamate residues to a small subpopulation of ribosomes (that are present at a concentration two orders of magnitude greater than that of Rim proteins), a competing activity is required, and this is provided by RimB. Proteolysis of RpsF glutamate tails by RimB and interaction between RimB and RimK serves to maintain the cellular ribosome population in a steady state of RpsF glutamylation.

Here we report a detailed investigation of the RimB protease and show it to be a rare and highly unusual bacterial example of a retroviral-like retropepsin of the A2 family of aspartic proteases. Biochemical analysis reveals the specificity determinants of RimB and optimal cleavage conditions for glutamate removal from polyglutamylated RpsF. We show that conservative mutation of the catalytic aspartate residue abolishes this activity. Furthermore, we demonstrate the bifunctional activity of RimB in stimulating poly-α-glutamate production by RimK *in vitro* and *in vivo* and show that this activity correlates with increased sensitivity to antibiotics. Finally, phylogenetic analysis infers that *rim* gene distribution is not congruent with the evolution of the host organisms, establishing the Rim pathway as an accessory regulatory element.

## Results

### RimB glutamate removal activity is favored at high pH and is insensitive to common inhibitors

**Figure 1:**
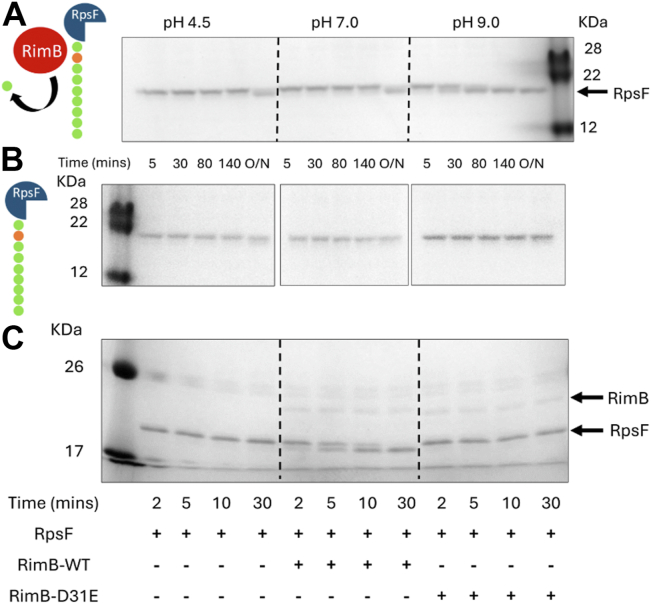
**RimB proteolysis of the RpsF C-terminal tail is favoured at high pH and requires conserved D31.***A*, the rate of RpsF proteolysis increases significantly at alkaline pH. 12% SDS-PAGE gel showing differential rate of RpsF cleavage according to pH. RpsF-Cmix6 was present at a concentration of 12.0 μM and is visible in each lane of the gel. RimB was present at a concentration of 0.23 μM. RimB is not visualised at this concentration. Cleavage of RpsF is represented by the disappearance of the original RpsF band and the appearance of a new lower molecular weight band. The pH and time course (in minutes or O/N – 16 h) of each digestion is indicated. *B*, in the absence of RimB, only minor degradation of RpsF occurs following overnight incubation at pH 4.5. *C*, 12% SDS-PAGE gel showing the essentiality of RimB residue D31 for catalytic function. SBW25 RpsF with a modified C-terminus possessing the additional residues EDEEEEEEEE was present at a concentration of 14 0.5 μM. Wild-type RimB and RimB-D31E were present where indicated at a concentration of 4.8 μM. The experiment was conducted at pH 9.0. Sampling times are as given.

The proteolytic activity of RimB was assessed *in vitro* using RpsF variants engineered with cleavable C-terminal tails comprising 10 additional acidic residues (see Materials and Methods). Samples were incubated at pH 4.5, 7.0 or 9.0. A catalytic quantity of RimB (diluted at the appropriate pH) was introduced and samples were withdrawn for SDS-PAGE at regular intervals. At pH 4.5 and 7.0, observable cleavage of the RpsF glutamate tail was only achieved following overnight incubation. However, digestion was significantly faster at pH 9.0 with cleavage completing between 80 and 140 min. ATP and glutamate were not required for proteolytic activity (Fig. 1*A*). In the absence of RimB, no cleavage of RpsF was observed in any condition tested (Fig. 1*B*).

**Figure S1:**
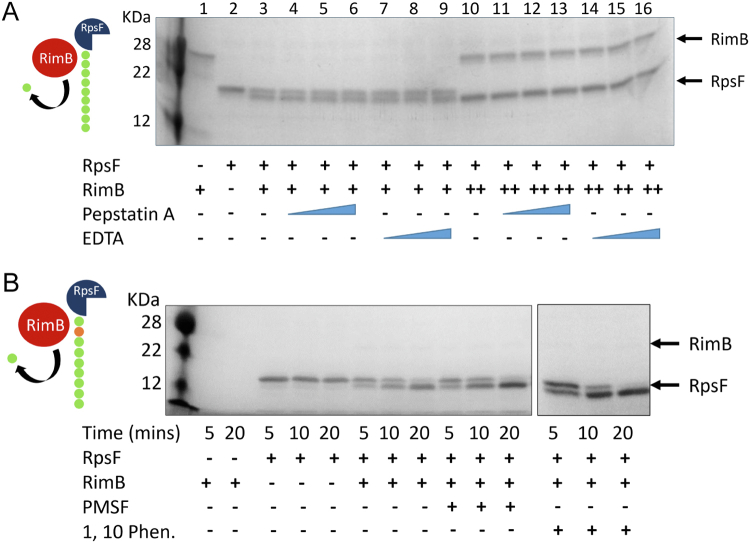


The apparent inactivity of RimB at low pH might suggest that the protein is a member of the alkaline protease family of enzymes containing the metalloproteases and serine proteases. Furthermore, BLAST analysis (32) of the RimB protein sequence (excluding the *Pseudomonas* genus) revealed a significant alignment to proteins annotated as zinc-dependent proteases. To investigate this possibility, the ability of RimB to cleave RpsF was tested in the presence of EDTA and 1,10-phenanthroline (inhibitors of metalloproteases) together with PMSF (an inhibitor of serine proteases) and pepstatin A (an inhibitor of aspartyl proteases). In all cases, the ability of RimB to proteolyse RpsF was unaffected (Fig. S1, *A* and *B*). Additionally, whilst RimB contains two cysteine residues, neither resides within a catalytic triad diagnostic for cysteine proteases (33). Thus, RimB eludes characterization as a member of a typical bacterial class.

### *rimB* can exist as a hybrid gene with *rimK* encoding a proteolytically active gene product

**Figure S2:**
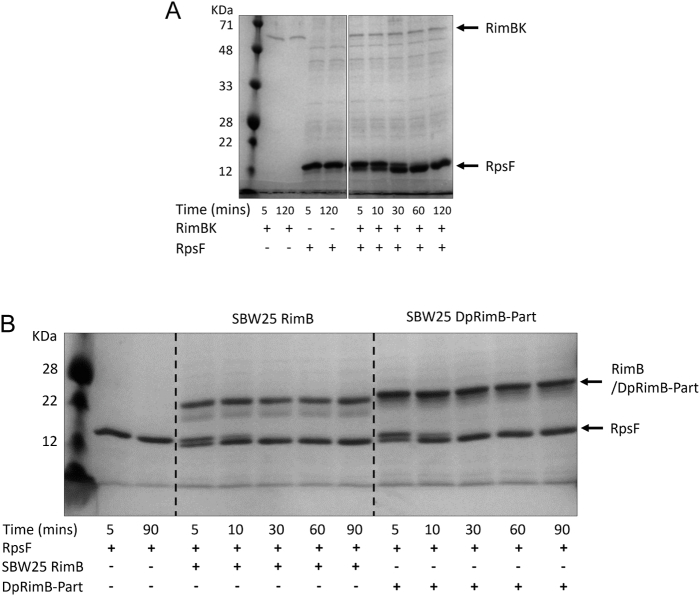


A preliminary BLASTp analysis of SBW25 RimB within the MEROPS database (5), revealed the existence of a putative hybrid protein containing a domain possessing primary sequence identity with RimB at the N-terminus of RimK; we term this protein RimBK. Purification of this hybrid protein from *Desulfotalea psychrophila* revealed it to possess protease activity against polyglutamylated RpsF (Fig. S2*A*). To confirm that the proteolytic activity of the RimBK protein was due to the RimB domain region, we purified the RimB portion of the enzyme encompassing residues 1 to 163. The isolated RimB domain was found to proteolyse glutamylated SBW25 RpsF with the same efficiency as the SBW25 RimB protein (Fig. S2*B*).

### RimB is a retropepsin-like aspartic endoprotease

We sought to further investigate the nature of RimB by performing homology detection and structure prediction using the HHpred Bioinformatics Toolkit (34). Remarkably, RimB was found to align with a family of retroviral and retroviral-like proteases; members of the A2 family of aspartic endoproteases. Interestingly, precedents of pepstatin-insensitive aspartic proteases have been described raising the possibility that RimB is such an example (35, 36, 37).

**Figure 2:**
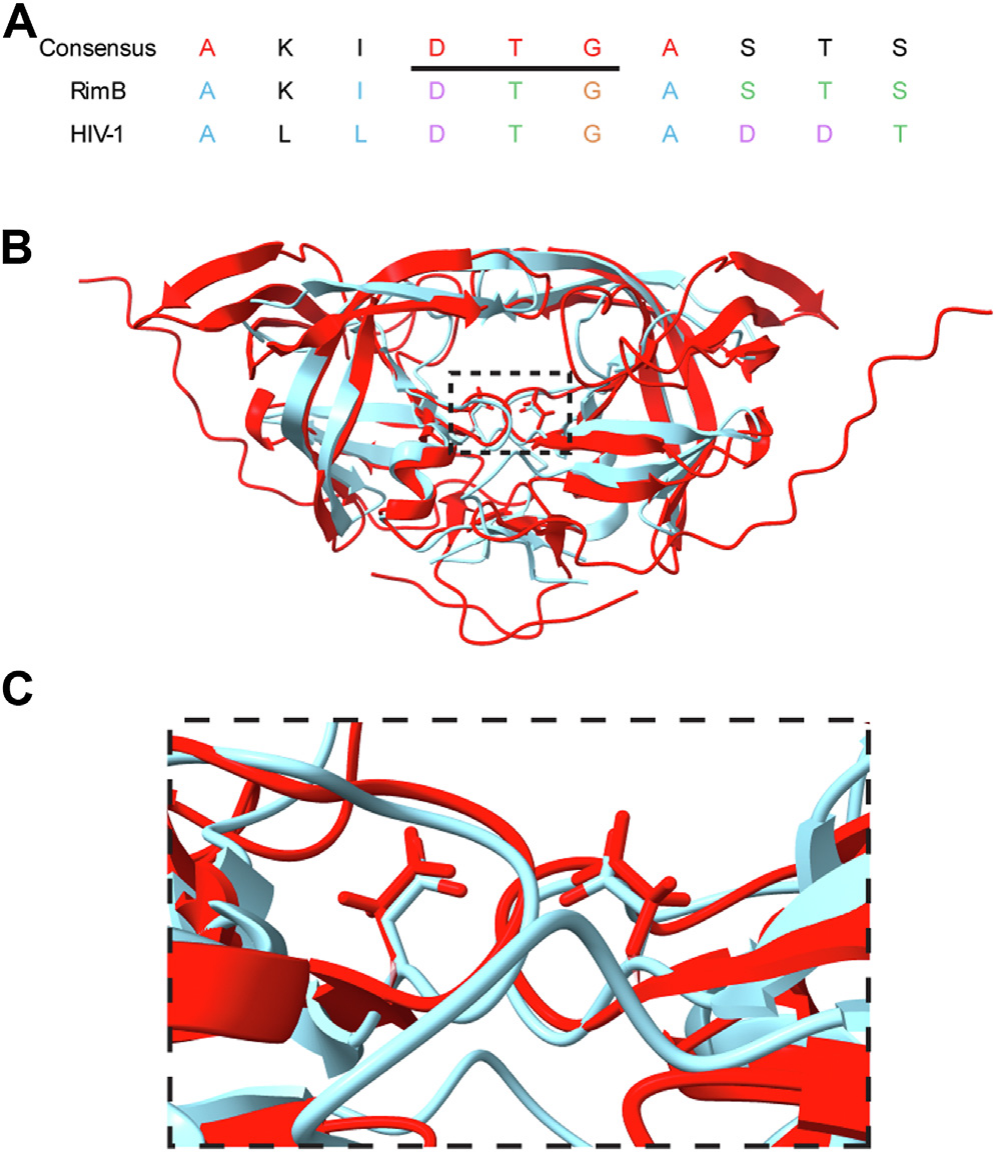
**RimB possesses the active site motif and significant tertiary structure conservation with HIV-1 protease.***A*, primary amino acid alignment. An alignment of RimB and HIV-1 protease (Uniprot identifier Q7SS10) showing RimB residues 28 to 37 and the corresponding HIV-1 identity. The DTG motif (underlined) that includes the active site aspartic acid is conserved. *B*, overlay of the cartoon representation of dimeric HIV-1 peptidase and RimB structures. *Cyan*: Crystallographic structure (2HS1) of the HIV-1 peptidase. *Red*: Predicted RimB structure predicted by AlphaFold (C3K4J0). *C*, expansion of the hatched area in Panel B showing the catalytic aspartate residue of each monomer as sticks for RimB (*red*) and HIV-1 (*cyan*).

Alignment of RimB with the HIV-1 protease (required for the maturation of the HIV virion) revealed a degree of primary amino acid identity around the wholly conserved DTG motif containing the catalytic aspartate in A2 family proteases and other aspartic proteases including pepsin (Fig. 2*A*). Significantly, a comparison of the HIV-1 dimeric crystallographic structure with the predicted RimB structure generated by AlphaFold (38) revealed structural similarities including the putative position and orientation of the catalytic aspartate side chain (Fig. 2, *B* and *C*). This catalytically competent dimer of RimB (whereby the catalytic aspartate residues from each monomer are brought into close proximity) can be convincingly modeled onto the RimK enzyme in an interaction that results in the upregulation of RimK ATPase activity (19, 31).

To confirm the essentiality of the conserved aspartate (D31) residue of RimB for proteolysis function, a substitution of this residue was made to produce the RimB D31E mutant protein. This conservative mutation of the catalytic aspartate residue has been shown to prevent autocatalysis through disruption of the hydrogen bonding network associated with the active site (39). Incubation of RpsF with wild-type RimB resulted in cleavage of RpsF within minutes at the given concentrations. Conversely, incubation of RpsF with an equivalent concentration of RimB D31E did not result in any detectable proteolysis over the same time frame, inferring that the catalytic activity of RimB D31E had been disabled (Fig. 1*C*).

**Figure S3:**
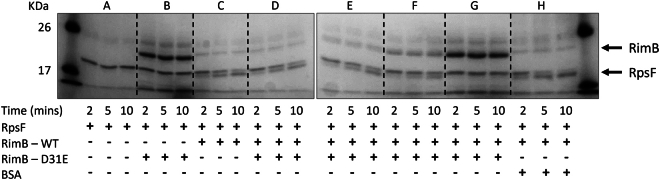


Next, we sought evidence that the inactivity of RimB D31E was not a consequence of protein instability by assessing the ability of the mutant to self-associate. We reasoned that homodimerisation of RimB D31E should not be affected provided that the integrity of the protein tertiary structure remained unaffected by the mutation. As previously demonstrated, incubation of RpsF with wild-type RimB resulted in C-terminal cleavage of RpsF (Fig. S3*C*), while incubation with a high concentration of RimB D31E did not result in proteolysis (Fig. S3*B*). Increasing concentrations of RimB D31E progressively inhibited the ability of wild-type RimB to proteolyse RpsF (Fig. S3, panels *D*–*G*). A high concentration of BSA, however, exerted no effect on the activity of wild-type RimB. Thus, we conclude that RimB D31E titrates down the proteolytic activity of wild-type RimB, presumably as a consequence of the increasing presence of inactive heterodimers of wild-type RimB and RimB D31E.

### Recognition determinants for RimB proteolytic activity

To cleave carboxy-terminal glutamate residues from RpsF, RimB must recognize this region of the protein. Hence, RimB may feasibly interact with glutamate residues, the carboxy region of RpsF, or both. To address this question, numerous forms of RpsF-bearing mixed engineered C-terminal tails comprised of glutamate residues and aspartate residues and numbering 10 amino acids in total were purified. An all-aspartate tail was not apparently recognized as no visible cleavage took place during the time course of the experiment whilst an all-glutamate tail was rapidly proteolyzed (Table 1). Analysis of all constructs tested suggests that cleavage will take place when six or more contiguous glutamate residues are present, with the rate of proteolysis reducing in an approximately proportional manner to the number of glutamate residues available. Furthermore, five contiguous glutamate residues apparently suffice when the first of these is the terminal genetically encoded glutamate residue. The RpsF protein fold *per se* is not required as a recognition requirement by RimB, because modification of the similarly sized ribosomal protein RpsI with a C-terminal tail incorporating the final two genetically encoded residues (aspartate and glutamate) plus a hexa-glutamate tail was cleavable by RimB. Furthermore, the substitution of the final two genetically encoded residues of RpsF with lysine and arginine still permitted cleavage albeit at a reduced rate. Finally, *E. coli* RpsF that is not subject to proteolytic modification *in vivo* was readily cleaved by SBW25 RimB when purified with an EDEEEEEEEE tail.

**Table 1:** Rate of polypeptide tail proteolysis by SBW25 RimB

Protein	Identifier	SUBSTITUTIONS-ADDITIONAL C-TERM residues	Rate relative to RpsF-10Glu
SBW25 RpsF	**RpsF-10Glu**	DNADE**-EEEEEEEEEE**	**N/A**
SBW25 RpsF	**RpsF-10Asp**	DNADE**-DDDDDDDDDD**	**NIL**
SBW25 RpsF	**RpsF-1Glu, 9Asp**	DNADE**-EDDDDDDDDD**	**NIL**
SBW25 RpsF	**RpsF-2Glu, 8Asp**	DNADE**-EEDDDDDDDD**	**NIL**
SBW25 RpsF	**RpsF-3Glu, 7Asp**	DNADE**-EEEDDDDDDD**	**NIL**
SBW25 RpsF	**RpsF-Cmix4**	DNADE**-EEEDDEEEEE**	**NIL**
SBW25 RpsF	**RpsF-Extend7**	DNADE**-EEEEDDDDDD**	- - -
SBW25 RpsF	**RpsF-Cmix2**	DNADE**-EEEEDEEEDE**	- - -
SBW25 RpsF	**RpsF-Cmix1**	DNADE**-EEEEDEEEEE**	- - -
SBW25 RpsF	**RpsF-Extend11**	DNADE**-DDDDEEEEEE**	- -
SBW25 RpsF	**RpsF-Cmix3**	DNADE**-EEEDEEEEEE**	- -
SBW25 RpsF	**RpsF-Extend8**	DNADE**-EEEEEDDDDD**	-
SBW25 RpsF	**RpsF-Extend9**	DNADE**-DEEEEEEEEE**	-
SBW25 RpsF	**RpsF-Cmix6**	DNADE**-EDEEEEEEEE**	-
SBW25 RpsF	**RpsF-Cmix5**	DNADE**-EEDEEEEEEE**	-
SBW25 RpsF	**RpsF-Extend10**	DNADE**-DDDEEEEEEE**	-
SBW25 RpsF	**RpsF-KR-Cmix6**	DNA**KR-EDEEEEEEEE**	- -
SBW25 RpsF	**RpsF-E140K-10Glu**	DNAD**K-EEEEEEEEEE**	- -
SBW25 RpsF	**RpsF-D139K-10Glu**	DNA**K**E**-EEEEEEEEEE**	-
SBW25 RpsF	**RpsF-D139A-10Glu**	DNA**A**E**-EEEEEEEEEE**	-
SBW25 RpsF	**RpsF-E140D-10Glu**	DNAD**D-EEEEEEEEEE**	-
*E. coli* RpsF	***E. coli* RpsF-Cmix6**	GDSEE**-EDEEEEEEEE**	-
SBW25 RpsI	**RpsI-tail5**	**-DNADEEEEEEEEEEE**	-
SBW25 RpsI	**RpsI-tail3**	**-NADEEEEEEEEEEE**	**≡**
SBW25 RpsI	**RpsI-tail2**	**-ADEEEEEEEEEEE**	**≡**
SBW25 RpsI	**RpsI-tail1**	**-DEEEEEEEEEEE**	**≡**

In the penultimate column, the final five genetically encoded residues of the stated protein are shown as unhighlighted residues (prior to the hyphen preceding the added tail) unless one of these residues has been substituted in which case the mutated amino acid is highlighted in bold. The bold residues following the hyphen denote the added polypeptide tail. Glutamate tail residues are highlighted in green and aspartate tail residues are highlighted in orange. The final column shows the relative rate of proteolysis compared to RpsF-10Glu, indicated as follows: N/A – Not Applicable; NIL – no proteolysis observed throughout the time course of the experiment in which RpsF-10Glu is proteolyzed to its fullest extent; three dashes – very slow rate of proteolysis; double dash – slow rate of proteolysis; single dash – slightly reduced rate of proteolysis; equivalence symbol (triple stacked dash) – same rate as RpsF-10Glu.

### RimB stimulates poly-**α**-L-glutamate synthesis by RimK

**Figure 3:**
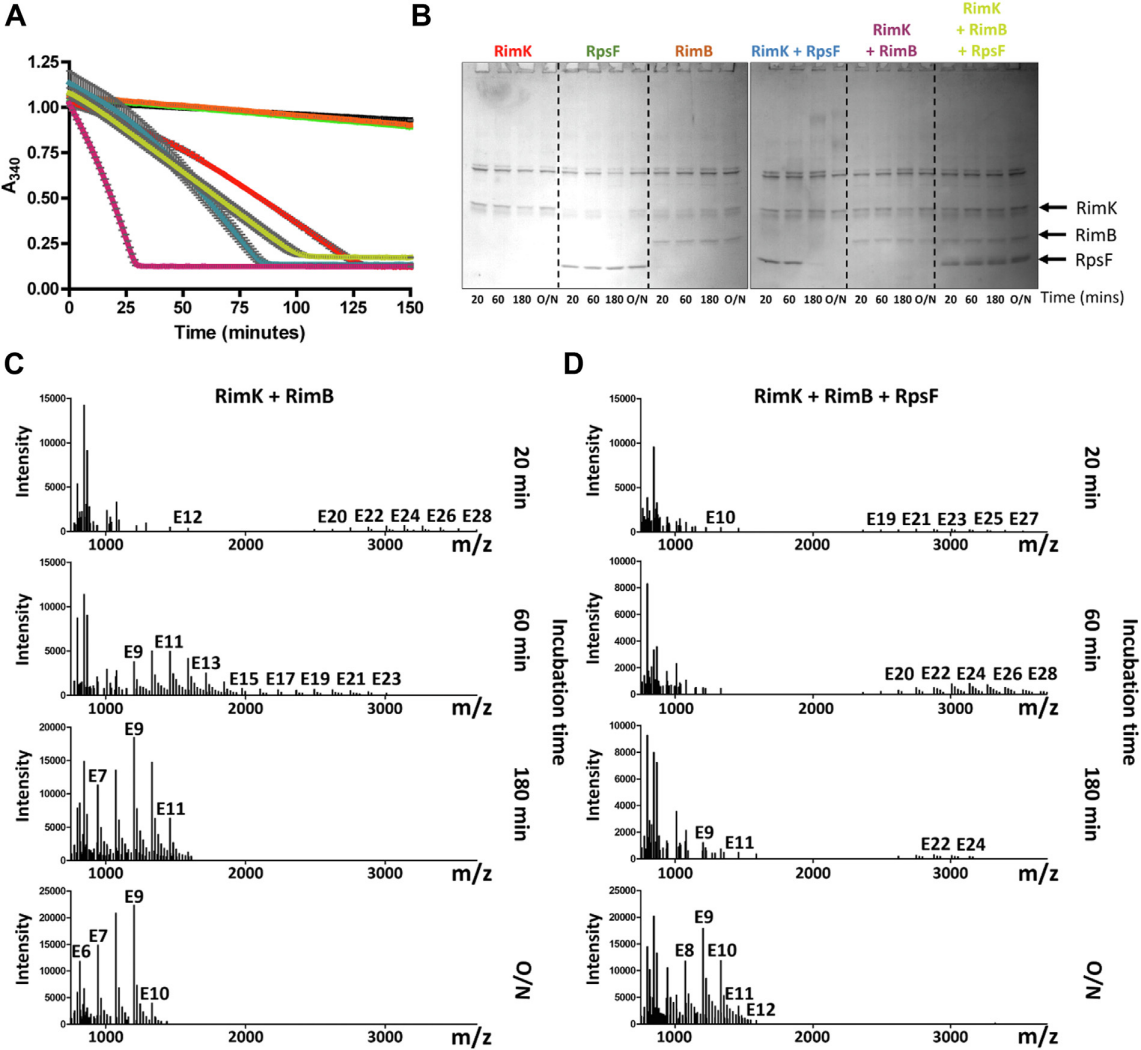
**SBW25 RimK synthesises poly-α-L-glutamate *in vitro* in the presence of RimB.***A*, ATPase activity assay showing the influence of SBW25 RimB and RpsF on RimK activity. SBW25 RimK was present at a concentration of 3.8 μM, RimB at 6.0 μM as indicated below and RpsF at 6.4 μM as indicated below. At 20 min, 60 min, 180 min and overnight (O/N), 20 μl was withdrawn for SDS-PAGE analysis and 40 μl withdrawn for polyglutamate detection. Each activity curve shows the average of duplicate samples. Error bars show the standard error of the mean. *Black line* – assay components only; *green line* – RpsF only; *orange line* – RimB only; *Red line* – RimK only (specific activity = 47 nmol/min/mg RimK); blue line – RimK + RpsF (specific activity = 77 nmol/min/mg RimK); purple line – RimK + RimB (specific activity = 196 nmol/min/mg RimK); *gold line* – RimK + RimB + RpsF (specific activity = 58 nmol/min/mg RimK). *B*, SDS-PAGE analysis (12%) of samples taken at the indicated time (as in *A*). The positions of RimK, RimB and RpsF within the gels are indicated by arrows. *C*, mass spectrometric detection of poly-α-L-glutamate polymers (as in *A*) synthesised in the presence of 3.8 μM SBW25 RimK and 6.0 μM SBW25 RimB. For consistency, all spectra were deconvoluted to show m/z for +1 charge. Each major peak differs by one glutamate residue (129 Da) with m/z values consistent with ionised polymers forming sodium adducts in the experiment. Numbers above a peak give the number of glutamate residues present in the polymer at that position. *D*, mass spectrometric detection of poly-α-L-glutamate polymers from *A* above, synthesised in the presence of 3.8 μM SBW25 RimK, 6.0 μM RimB and 6.4 μM RpsF. Each major peak differs by one glutamate residue with m/z values consistent with ionised polymers forming sodium adducts in the experiment. Numbers above a peak give the number of glutamate residues present in the polymer at that position.

RimK from *E. coli* is the product of a monocistronic gene and has been demonstrated to synthesize poly-α-L-glutamate *in vitro* in addition to its function as a glutamyl ligase enzyme (29). We wished to determine if RimK from *P. fluorescens* SBW25 was also competent to synthesize polypeptides of glutamate, and whether such activity was modulated by RimB. We have previously demonstrated that in addition to possessing glutamate residue removal activity, RimB stimulates the ATPase activity of RimK through direct interaction (19). Recent work by this laboratory has modeled the predicted interaction between the RimB and RimK enzymes from *P. aeruginosa* (31). Intriguingly, heightened RimK ATPase activity is not directly related to RpsF glutamylation activity suggesting an alternative function linked to ATP turnover (20). SBW25 RimK was incubated in the presence or absence of either SBW25 RimB, SBW25 RpsF, or both proteins. The ATPase activity of RimK was simultaneously measured (Fig. 3*A*) and samples were withdrawn after 20 min, 60 min, 180 min, and overnight for polyglutamate detection and SDS-PAGE analysis.

A modest stimulation of RimK occurred upon the addition of RpsF; the whole population of RpsF was glutamylated by RimK and shifted to high molecular weight within 180 min (Fig. 3*B*). Upon the addition of RimB to RimK, specific activity increased fourfold. In contrast, the additional presence of RpsF reduced the specific activity of RimK closer to that of RimK alone. Inspection of SDS-PAGE samples revealed that glutamylation of RpsF had effectively been prevented, presumably due to a degree of interaction between RimK and RimB (limiting the extent of interaction between RimK and RpsF) coupled with proteolytic activity of RimB on RpsF (20). The extent of poly-α-L-glutamate production in each of the samples was then assessed by mass spectrometry. Interestingly, detectable levels of polyglutamate were only found in samples containing both RimK and RimB. In the presence of RimB, a distribution of glutamate polypeptides was resolved within the first 20 min with a maximum chain length of 28 glutamate units (Fig. 3*C*). Degradation of the polypeptides subsequently occurred coincident with exhaustion of the NADH electron donor present in the coupled ATPase assay. Interestingly, a similar polypeptide distribution was recovered from the sample containing RimB and RpsF (Fig. 3*D*). However, the formation of polyglutamate was slower. Once again, the products were degraded at approximately the same time that ATP was no longer regenerated.

We reasoned that poly-α-L-glutamate stability may be dependent upon solute pH. To investigate this possibility, poly-α-L-glutamate was synthesized by incubating RimK and RimB under standard conditions at pH 9.0. RimK ATPase activity was simultaneously measured, and the reaction was quenched when ATP regeneration ceased to ensure maximal production. Poly-α-L-glutamate was purified from the reaction in three equal volumes and subsequently resuspended into a buffer at pH 4.5, 7.0, or 9.0. Following incubation for 1 h at room temperature, poly-α-L-glutamate was subsequently re-purified from the samples and submitted for mass spectrometry. In this instance, a maximum chain length of 36 glutamate units was resolved at pH 9.0 however polymer chain length and apparently abundance of the poly-α-L-glutamate product reduced with decreasing pH (Fig. S4). This would suggest that poly-α-L-glutamate stability is favored at high pH.

**Figure 4:**
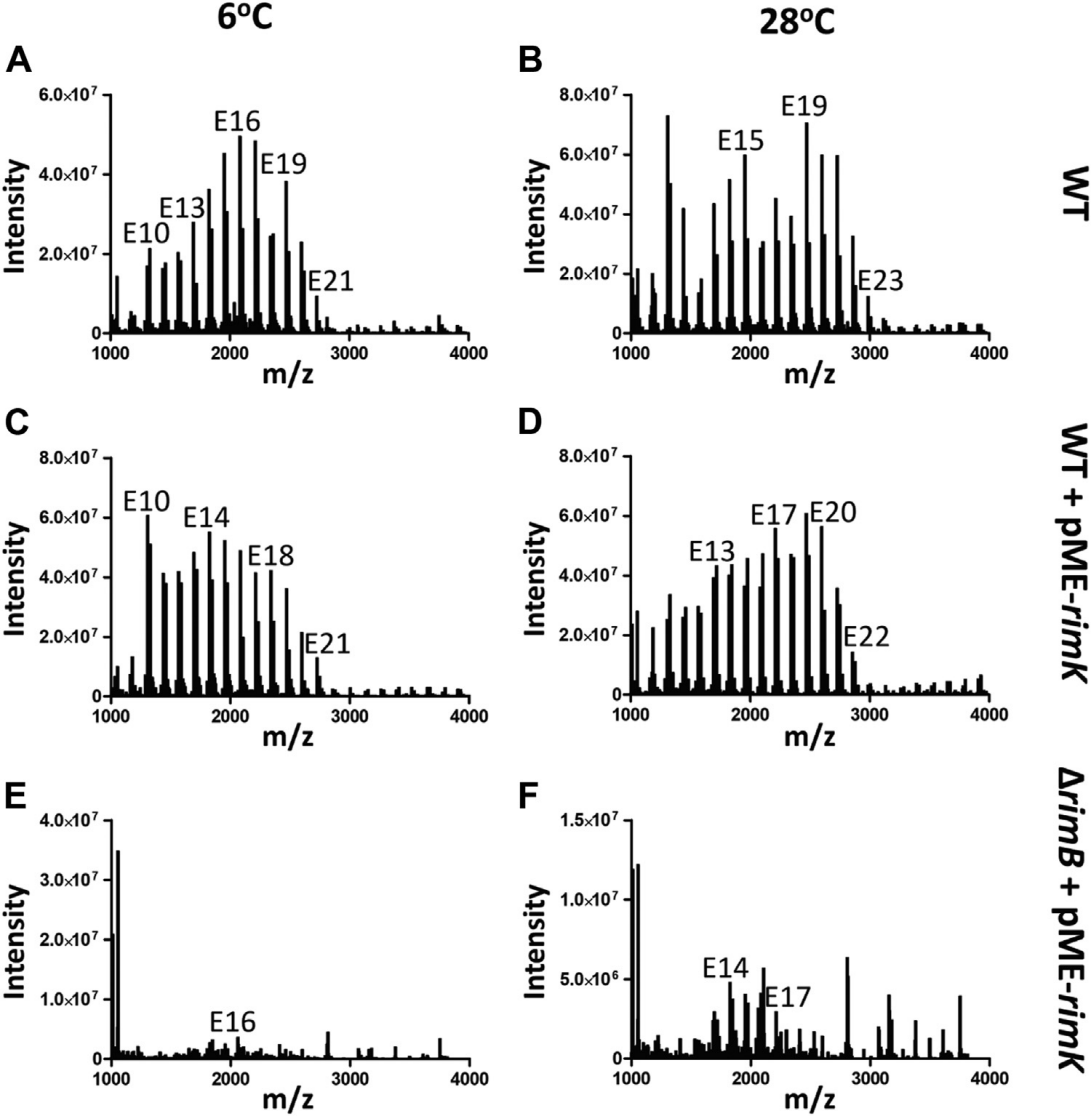
**SBW25 RimK synthesises poly-α-L-glutamate *in vivo*.** SBW25 cells were grown at the indicated temperature in M9-0.4% Pyruvate media at an agitation rate of 250 rpm. The cells were pelleted when an optical density (measured at 600 nm) reached 0.3. Poly-α-L-glutamate polymers were detected by MS, as in Figure 3. *A* and *B*, SBW25 wild-type cells. *C* and *D*, SBW25: pME-*rimK* (*rimK* overexpression strain). *E* and *F*, SBW25: Δ*rimB*, pME-*rimK* (*rimK* overexpression in a Δ*rimB* background).

Having established the ability of RimK to synthesize poly-α-L-glutamate *in vitro* and identified RimB as a catalytic factor in stimulating production, we sought evidence for this activity *in vivo*. Recovery from wild-type cells grown to mid-exponential phase in M9-Pyruvate media at 6 °C or 28 °C was typically low, indicating a low level of free polypeptide *in vivo*. That said, a reproducible distribution of polypeptide chains was resolved, trending towards higher molecular weight with increasing temperature yet with a maximal length slightly shorter than that typically recovered *in vitro* (Fig. 4, *A* and *B*).

To further investigate the relationship between RimB and poly-α-L-glutamate synthesis *in vivo*, RimK was overexpressed in SBW25 wild-type cells and in an SBW25 Δ*rimB* mutant strain. In contrast to the wild-type strain overexpressing RimK that produced readily purifiable polypeptides (Fig. 4, *C* and *D*), a low signal-to-noise ratio complicated identification of poly-α-L-glutamate peptides from the mutant strain, especially in cells grown at 6 °C (Fig. 4, *E* and *F*). This suggests that levels of poly-α-L-glutamate were very low in the mutant and supports the notion that RimB is an essential factor in promoting efficient poly-α-L-glutamate synthesis by RimK.

### Overexpression of SBW25 RimK increases antibiotic sensitivity in a RimB-dependent manner and independently of ribosomal modification

**Figure 5:**
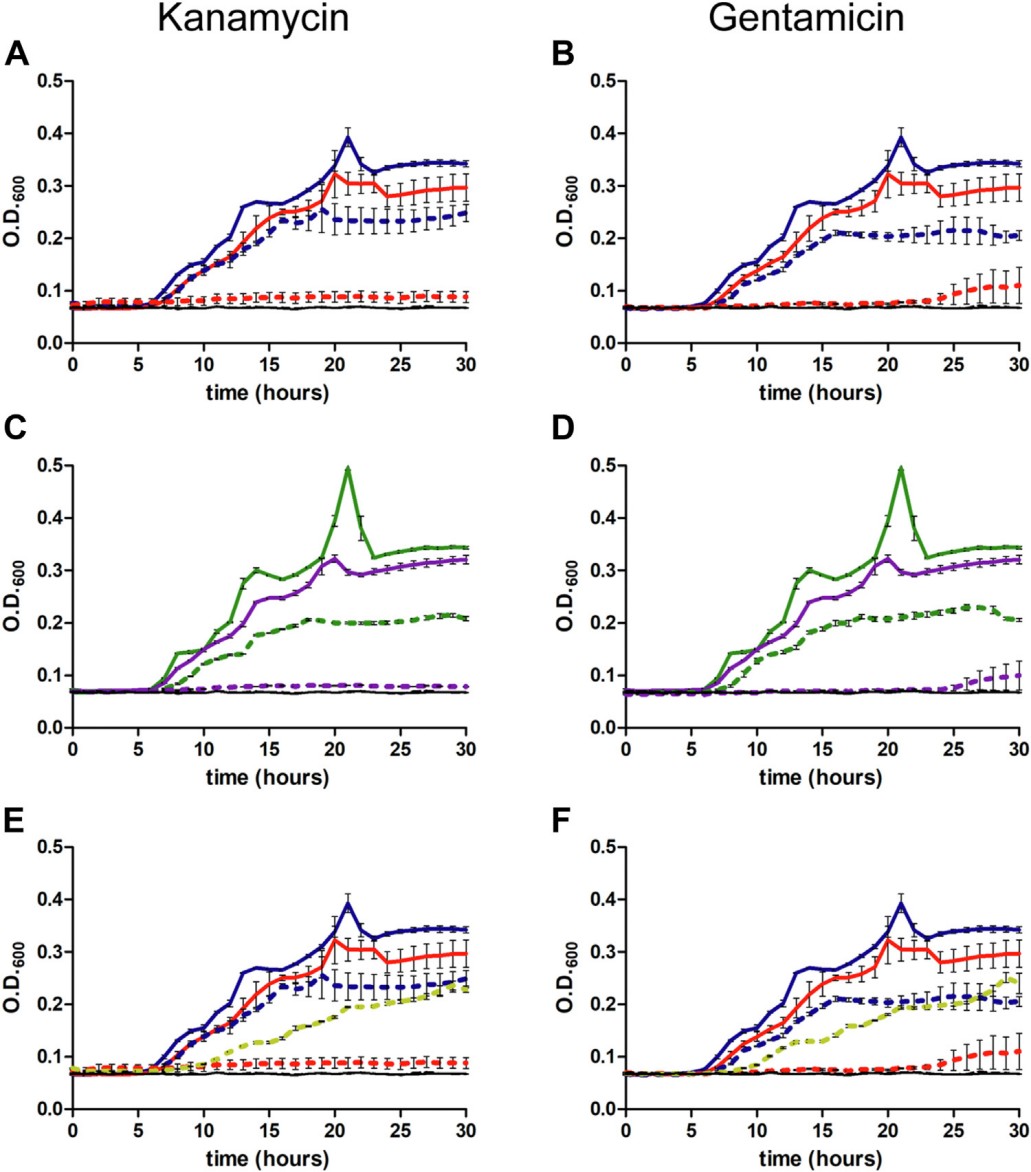
**Influence of RimK overexpression on antibiotic sensitivity.***A*, SBW25 cells were grown in the absence (*solid lines*) or presence (*dashed lines*) of the indicated antibiotic. In all panels, the *black* baseline data represents the absorbance of the growth media alone during the experiment. *A* and *B*, *blue*– wild-type cells with pME6032 empty vector; *red*– wild-type cells with pME-*rimK*. *C* and *D*, *green*– SBW25 *rpsF-D139K* cells with pME6032 empty vector; *purple* - SBW25 *rpsF-D139K* cells with pME-*rimK*. *E* and *F*: *blue*– wild-type cells with pME6032 empty vector; *red*– wild-type cells with pME-*rimK*; gold - SBW25 Δ*rimB* cells with pME-*rimK*. Kanamycin was used at a concentration of 0.75 μg/ml and gentamicin was used at a concentration of 0.4 μg/ml. Error bars show the standard error of the mean. The data in all panels was acquired as a single growth experiment and has been separated here for clarity.

We reasoned that Rim-dependent modification of ribosomes may alter the response of SBW25 to aminoglycoside antibiotics. To address this question SBW25 wild-type and mutant strains were grown at 28 °C in a rooting solution containing 0.4% w/v sodium pyruvate and varying concentrations of antibiotic. In the presence of kanamycin or gentamicin, cells overexpressing RimK were noticeably more sensitive, displaying greatly attenuated growth (Fig. 5, *A* and *B*). To ascertain whether the observed increase in susceptibility was due to ribosomal modification at RpsF, we included a mutant of SBW25 encoding a non-modifiable form of RpsF, RpsF-D139K (19). To our surprise, overexpression of RimK in the *rpsF-D139K* genetic background resulted in the same hypersensitivity to either kanamycin or gentamycin (Fig. 5, *C* and *D*).

Thus, we concluded that increased sensitivity to aminoglycoside antibiotics resulting from overexpression of RimK was due to some other function of the protein unrelated to direct ribosome modification. Having established that RimK exhibits bifunctionality by glutamating RpsF and synthesizing poly-α-L-glutamate peptides, we considered that a link might exist between polypeptide production by RimK and antibiotic sensitivity. As we had established that RimB is a stimulatory factor in RimK-catalysed polypeptide production, we predicted that removal of RimB would lessen growth inhibition in the presence of antibiotics if poly-α-L-glutamate was the root cause of increased sensitivity. Indeed, overexpression of RimK in the Δ*rimB* genetic background resulted in a significant recovery of antibiotic resistance (Fig. 5, *E* and *F*).

### The ATPase activity of *E. coli* RimK is not responsive to the presence of SBW25 RimB

Having established that RimB does not recognize genetically encoded protein elements when cleaving post-translationally added glutamate residues, we were interested to know whether the ATPase activity of RimK from *E. coli* (that does not possess a copy of *rimB*) possessed the ability to be stimulated by SBW25 RimB. In SBW25, upregulation of RimK ATPase activity correlates with enhanced poly-α-L-glutamate production. RimK from *E. coli* has been shown to possess intrinsic poly-α-L-glutamate synthesis activity but a lack of *rimB* infers that the rate of synthesis may be invariable (29).

**Figure 6:**
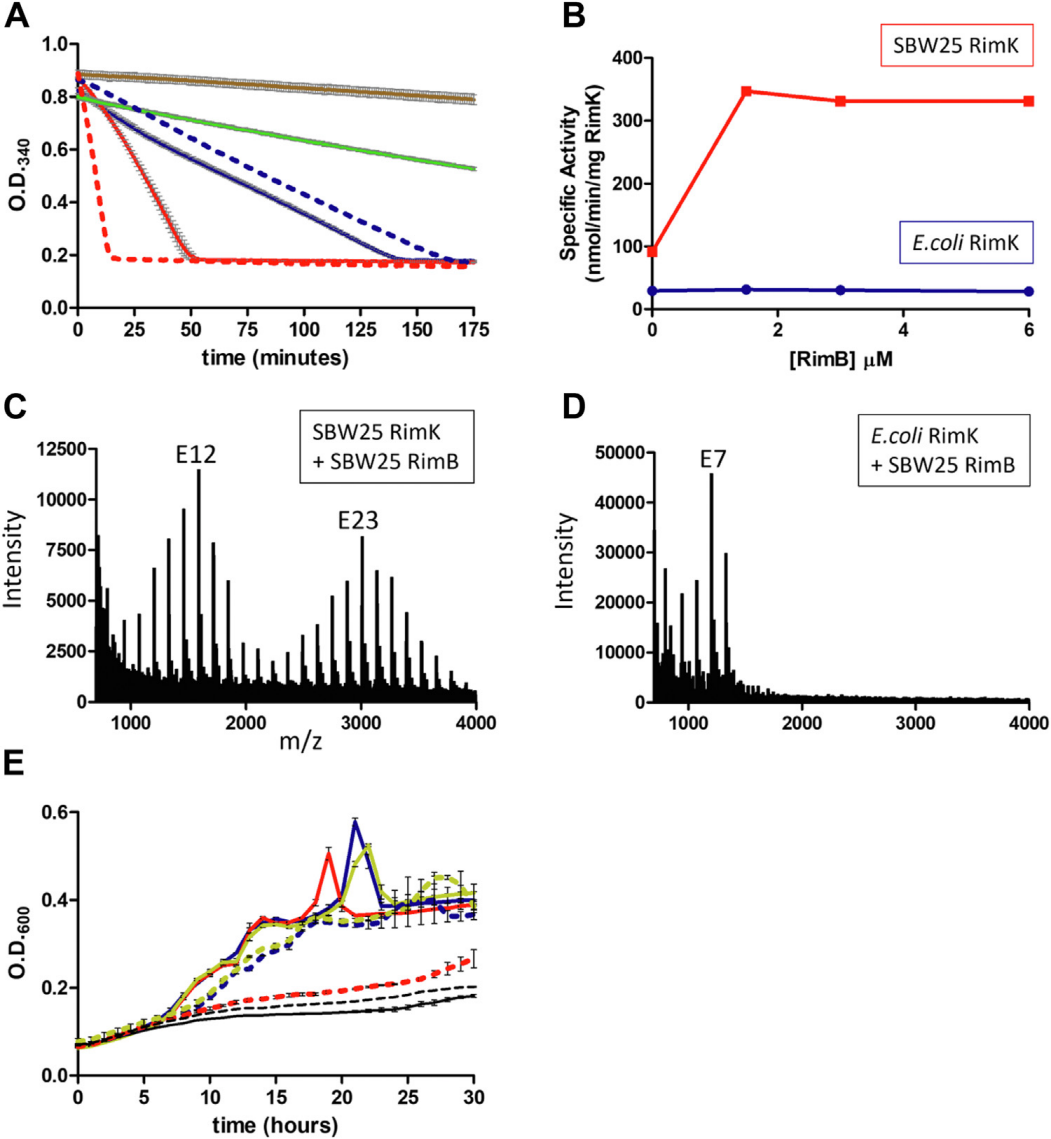
**ATPase and polyglutamate synthase activity of *E. coli* RimK is not stimulated by RimB.***A*, ATPase activity assay showing an example of the raw data. RimK proteins were present at a concentration of 3.8 μM. ATPase activity was measured in the absence (solid lines) or presence (dashed lines) of SBW25 RimB at a concentration of 6 μM. *Red*– SBW25 RimK; *blue*– *E. coli* RimK; *green*– SBW25 RimB alone; *brown*– buffer only. Error bars show the standard error of the mean. *B*, specific activity of RimK proteins measured in the presence of increasing concentrations of SBW25 RimB. *Red*– SBW25 RimK; *blue*– *E. coli* RimK. *C*, mass spectrometric detection of poly-α-L-glutamate polymers synthesised in the presence of 3.8 μM SBW25 RimK and 1.5 μM SBW25 RimB. Each major peak differs by one glutamate residue. The position of a 12-mer and 23-mer of polyglutamate is indicated above the respective peak. Samples containing 3.8 μM SBW25 RimK with either 3.0 μM or 6.0 μM SBW25 RimB were also acquired and gave the same overall result (data not shown). *D*, mass spectrometric detection of poly-α-L-glutamate polymers synthesised in the presence of 3.8 μM *E coli* RimK and 1.5 μM SBW25 RimB. Spectra were processed as described in Figure 3. The position of a 7-mer of polyglutamate is indicated above the respective peak. Samples containing 3.8 μM SBW25 RimK with either 3.0 μM or 6.0 μM SBW25 RimB were also acquired and gave the same overall result (data not shown). *E*, SBW25 Δ*rimK* cells were grown in the absence (solid lines) or presence (*dashed lines*) of the indicated antibiotic. The *black* baselines represent the absorbance of the growth media alone during the experiment. *Blue*– SBW25 Δ*rimK* cells with pME6032 empty vector; *red*– SBW25 Δ*rimK* cells with (SBW25) pME-*rimK*; *gold*– SBW25 Δ*rimK* cells with (*E. coli*) pME-*Ec-rimK*. Kanamycin was used at a concentration of 0.6 μg/ml. Error bars show the standard error of the mean.

An *in vitro* ATPase assay was performed to assess the activity of RimK proteins from SBW25 and *E. coli* and samples were taken as indicated to test for the presence of poly-α-L-glutamate. The intrinsic ATPase activity of SBW25 RimK at [3.8 μM] was greater than that of *E. coli* RimK at the same concentration (Fig. 6, *A* and *B*). It is unclear if this reflects a true difference in basal activity or is an artifact of purification. Nevertheless, upon the addition of RimB to SBW25 RimK, a distribution of poly-α-L-glutamate chains was readily resolved commensurate with upregulated ATPase activity as seen previously. However, *E. coli* RimK ATPase activity was unresponsive to the presence of RimB with only short-chain poly-α-L-glutamate peptides detected following overnight incubation suggesting that any polyglutamate synthesized was either of low molecular weight or rapidly degraded (Fig. 6, *C* and *D*).

Multiple copies of *E. coli rimK* result in increased glutamylation of RpsF in *E. coli* cells, indicative of increased activity (28). We reasoned that if the polyglutamate synthase activity of *E. coli* RimK was increased sufficiently by overexpression in SBW25 cells, increased antibiotic sensitivity would result. Thus, *E. coli rimK* was overexpressed in SBW25 cells deleted for *rimK* and in the absence and presence of kanamycin (Fig. 6*E*). Overexpression of SBW25 *rimK* in the *rimK*-deleted background again resulted in increased sensitivity to the antibiotic (Fig. 5, *A* and *B*). However, heterologous expression of *E. coli rimK* in this background did not affect susceptibility to kanamycin. This may suggest that in the absence of any interaction with RimB, that poly-α-L-glutamate synthesis was insufficient to produce the hypersensitive response to antibiotic.

### Introduction of a contiguous internal polyglutamate motif is sufficient to target proteins for proteolysis by RimB *in vitro* and *in vivo*

**Figure 7:**
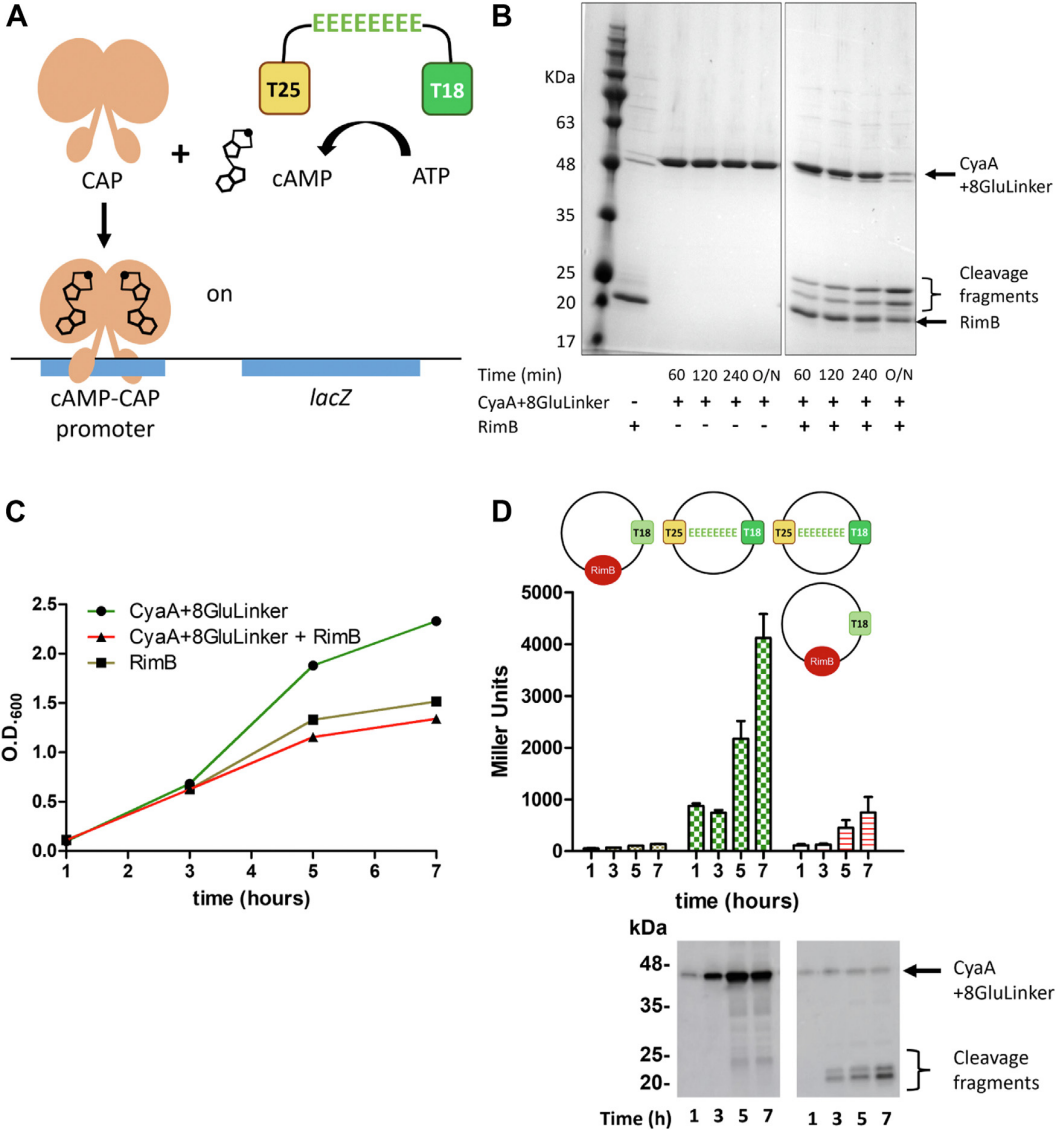
**Targeted protein cleavage by RimB.***A*, cartoon representation of a hybrid target protein formed by joining the catalytic domains of adenylate cyclase by an eight-glutamate linker and a cartoon representation of *lacZ* transcription resulting from a functional hybrid protein. The fusion of adenylate cyclase catalytic domains by an eight-glutamate motif results in the production of cyclic AMP (cAMP) and consequent activation of the catabolite activator protein (CAP). *B*, 12% SDS-PAGE gel showing the progressive cleavage of the hybrid protein in the presence of RimB. The hybrid protein was present at a concentration of 2.8 μM. RimB was present at a concentration of 24 μM. The assay was performed at pH 9.0. *C*, growth of BTH101 *E. coli* cells expressing RimB (*gold*), the hybrid target protein (*green*) or both proteins (*red*). Cells were grown in LB media at 28 °C. At the indicated times, the optical density of the samples was measured at 600 nm prior to assessing the β-galactosidase activity present in the sample. The cells were grown in the presence of 500 μM IPTG. *D*, β-galactosidase activity of BTH101 *E. coli* cells expressing RimB (*gold*), the hybrid target protein (*green*) or both proteins (*red*). Error bars show the standard deviation in each case. Anti-CyaA immunoblots are shown in the panels below. Each immunoblot lane corresponds to the bar above.

Having established that RimB can recognise a polyglutamate motif for proteolysis when present at the C-terminus of a non-canonical substrate (Table 1) we speculated that RimB might also proteolyse a sequence of glutamate residues that are internal to a protein sequence. To investigate this possibility, we fused the catalytic domains (T18 and T25) of adenylate cyclase from *Bordetella pertussis* (40) by introducing an eight-glutamate residue sequence between the two domains (Fig. 7*A*). Following purification of the hybrid protein, incubation in the presence of RimB resulted in the scission of the target *in vitro* into two cleavage fragments. In the absence of RimB, the target protein remained stable and intact (Fig. 7*B*).

Having established that RimB can cleave internal glutamate residue motifs *in vitro*, we wondered whether target recognition might be sufficient to allow RimB to perform the same reaction *in vivo*. Catalytic domains (T18 and T25) of adenylate cyclase linked by an eight-glutamate residue sequence were cloned into plasmid pKNT25 and *rimB* was cloned into the compatible T18 plasmid (Euromedex) (40). Assuming the catalytic domains are in appropriate proximity, cyclic-AMP (cAMP) will be synthesized allowing β-galactosidase production to be measured. Subsequent scission of the hybrid protein will prevent activity due to the separation of the subunits (Fig. 7*A*).

Growth of the reporter strain BTH101 Δ*cyaA* (Euromedex) overexpressing *rimB*, the hybrid target protein, or both proteins in LB was similar albeit the strain expressing the hybrid protein grew to a slightly greater optical density (Fig. 7*C*). As expected, expression of RimB alone produced only basal levels of β-Galactosidase activity (Fig. 7*D*). Expression of the hybrid protein alone resulted in significant β-Galactosidase activity that increased during the period of cell growth (Fig. 7*D*). The full-length hybrid protein was also confirmed by anti-CyaA immunodetection (Fig. 7*D*). In contrast, co-expression of both RimB and the hybrid target protein resulted in a significant reduction of adenylate cyclase activity (Fig. 7*D*). Immunodetection revealed that the hybrid target had been substantially cleaved into its’ constituent fragments (Fig. 7*D*).

### Bioinformatic identification of polyglutamate sequences present in bacterial genes

Having identified RimB as a specific protease of external and internal polyglutamate protein sequences, we were interested to know if bacteria possessing *rimB* contained genes encoding polyglutamate residue sequences within their genomes. Accessions containing *rimBK* were included in the analysis.

**Figure 8:**
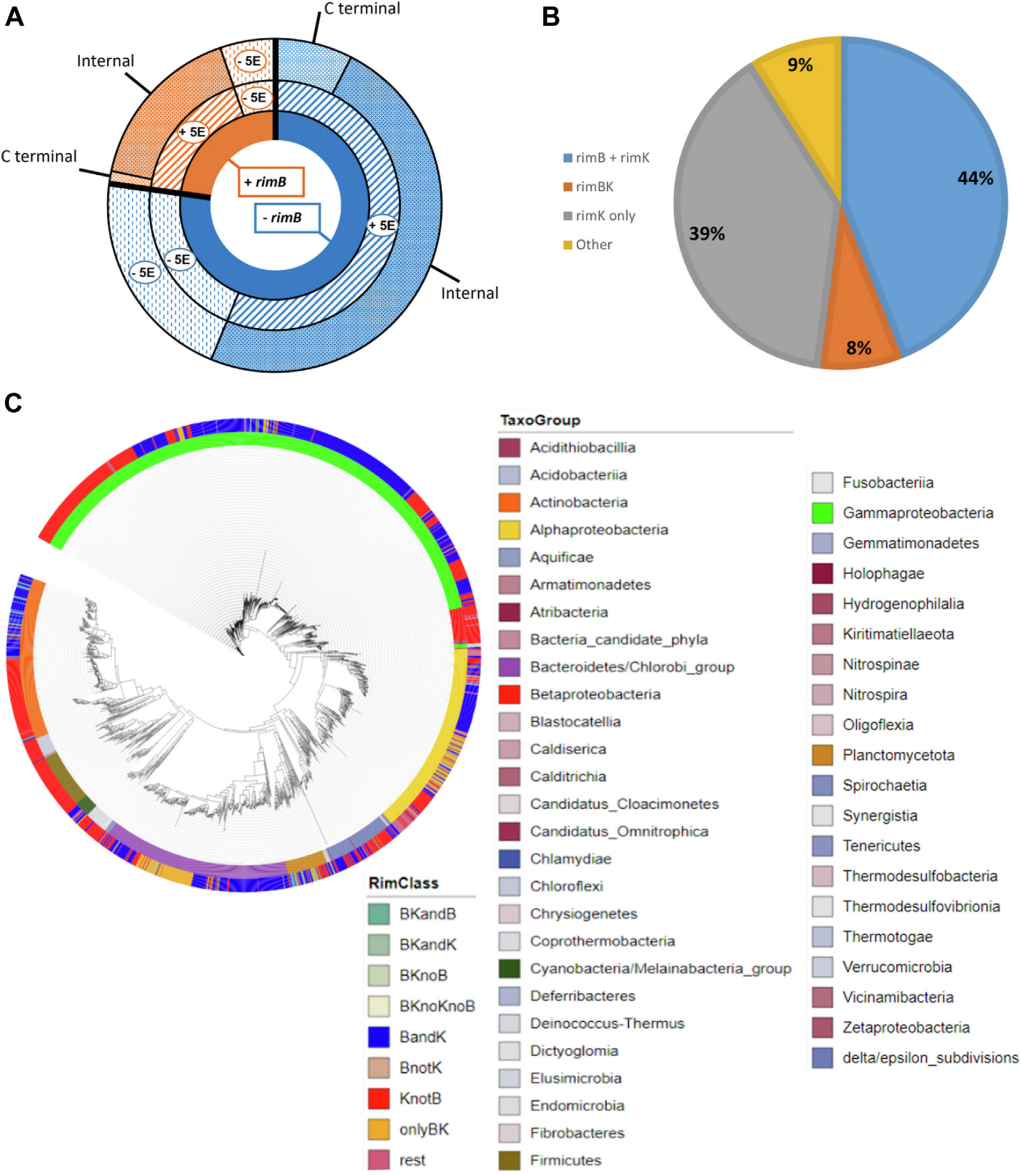
**Occurrence of *rim* genes in bacteria.***A*, pie chart representation showing the distribution of 12,706 bacterial genes classified according to gene arrangement. The *blue* region represents the 77% of bacteria that lack a *rimB* or *rimBK* gene (*inner circle*, *blue* region). Of these, 73% contain at least one gene encoding a minimum of five contiguous glutamate codons (*middle circle*, *blue* region labelled ‘+ 5E’). Of these, 13.5% encode at least one gene where the contiguous glutamate codons are located at the extreme C-terminus of the gene (*outer circle*, *blue* region). The orange region represents the 23% of bacteria that possess a *rimB* or *rimBK* gene (*inner circle*, *orange* region). Of these, 77% contain at least one gene encoding a minimum of five contiguous glutamate codons (*middle circle*, *orange* region labelled ‘+ 5E’). Of these, 7.4% encode at least one gene where the contiguous glutamate codons are located at the extreme C-terminus of the gene (*outer circle*, *orange* region). *B*, pie chart representation showing the *rim* gene organisation within 2226 bacterial accessions. Rare co-occurrences of *rimB* or *rimK* genes with *rimBK*, together with other sets that do not conform with the other major groups are collated under ‘Others.’ *C*, phylogenetic tree of the 2226 bacterial accessions containing *rim* genes. The inner coloured ring shows bacterial accessions coloured according to taxonomic group. The outer coloured ring shows bacterial accessions coloured according to *rim* genes encoded in the chromosome.

Surprisingly, 77% of accessions contained both *rimB* or *rimBK* and at least one gene encoding five or more contiguous codons encoding glutamate residues (Fig. 8*A*). This compares with 73% of accessions lacking a *rimB* or *rimBK* gene that possess at least one gene encoding five or more contiguous glutamate codons. Therefore, the possibility exists that additional gene products may be targeted by RimB for proteolysis. However, those accessions lacking *rimB* or *rimBK* possessed almost double the percentage of bacteria with at least one gene encoding five or more glutamate residues at the extreme C-terminus of the gene within their genome (Fig. 8*A*).

### Phylogenetic analysis of *rimB* and *rimK*

We next sought to investigate the co-occurrence of *rimB* with *rimK* and the degree of congruence with the relative phylogeny of the host organisms. 5959 bacterial accessions were searched by reciprocal BLASTp using RimB (*PFLU_0262*; UniProtKB Identifier C3K4J0), RimK (*PFLU_0261*; UniProtKB Identifier C3KE61) and the highest scoring RimBK hybrid protein from *D. psychrophila* LSv54 resulting from the RimB BLAST (*DP2392*; UniProtKB Identifier Q6AKK4). 37% (2226) accessions possessed at least one *rim* gene. Of these accessions, 44% contained *rimK* and *rimB* as distinct (non-hybrid) genes. While 12% contained *rimBK* (Fig. 8*B*). Of these, 2.8% possessed *rimBK* plus an additional copy of *rimB*, and 0.9% possessed *rimBK* with an additional copy of *rimK*. Interestingly, of these genes, 136 *rim* proteins, from 102 accessions, were found to be encoded within five genes of a potential mobile genetic element. A phylogenetic tree of *rim*-containing genes based on a concatenation of seven protein sequences (as described in Materials and Methods) was then constructed (Fig. 8*C*). The class of *rim* genes possessed by each organism was superimposed on the taxogroup of that organism to understand the relationship between *rim* gene class and phylogeny.

The *rimBK* gene encoding the hybrid RimBK protein was constrained to a relatively small number of bacterial Orders with the majority found within the Flavobacteriales and Rhodobacterales. Bacteria within these Orders are largely found in aquatic and commonly oceanic environments (41, 42). Conversely, those bacteria containing both *rimB* and *rimK* as distinct genes revealed a scattered distribution relative to phylogeny suggesting that the acquisition of this *rim* gene arrangement is unrelated to the evolution of the recipient relative to the donor. Interestingly, those bacteria containing only a *rimK* gene (therefore lacking a RimB-encoding sequence either as a hybrid or single gene) also revealed a widespread but less haphazard distribution than those accessions containing both *rimK* and *rimB* genes. Bacteria containing only *rimK* were strongly represented in the Enterobacterales, Streptomycetales, and Bacillales Orders.

## Discussion

Aspartic peptidases have, until recently, been thought to be restricted to eukaryotes and viruses. The prevailing dogma states that eukaryotic pepsin-like family A1 proteases gave rise to family A2 viral retropepsins or *vice versa*. However, the presence of both of these families in bacteria challenges this hypothesis (7, 10, 43). The presence of aspartic peptidases in bacterial cells mostly appears to be highly restricted, albeit new examples doubtless await discovery; a process potentially hampered by mis-annotation. Interestingly, bacterial homologs of pepsin-like proteins are prevalent in oceanic bacteria and plant symbionts (7). Whilst a lack of confirmed identification precludes definitive statements regarding the distribution of bacterial retropepsin-like proteases, the presence of such proteins appears to show some correlation with extreme aquatic environments or association with higher plants and animals including humans.

Here, we show that RimB is a retropepsin-like protease with a unique specificity to a polyglutamated substrate. Why should poly-α-L-glutamylation serve as an appropriate regulator of protein function? Potentially, the introduction of a surface-exposed region of negative charge will alter the electrostatic association of the target with other regions within the protein or with interacting partners (15, 44). The physiochemical properties of L-glutamate and its polymer form bestow characteristics on the modified target that cannot be reproduced with other charged amino acids. For example, the short distance between the side chain carbonyl oxygen atom and the main chain hydroxyl oxygen of glutamate contributes to the retention of cationic species (45). Furthermore, the relative rigidity of poly-α-glutamate may favor exosite architecture and provide an optimal docking interface for protein interaction, including proteolysis (46). Interestingly, it has been argued that the HIV–1 retroviral protease recognizes shape rather than a specific amino acid sequence (47).

Conversely, our results suggest that the poly-α-L-glutamate tail is the sole factor required for RimB recognition. A contiguous chain of five glutamate residues suffices for RimB proteolysis if these residues immediately follow the C-terminus of the protein. It is reasonable to assume that those residues closest to the RpsF protein fold will possess a degree of rigidity greater than that of distal residues that would be expected to be more disordered. Thus, we conclude that the inherently ordered nature of a small number of glutamate residues immediately abutting a protein fold has the potential for recognition by RimB.

We have demonstrated here that RimB interacts with RimK to stimulate the synthesis of poly-α-L-glutamate. Recent work detailing potential RimB-RimK interaction determinants and structural polymorphism within RimK provide a basis for understanding the mechanism by which RimK directs poly-α-L-glutamate synthesis for ribosomal modification or polypeptide production (31, 48). The distinct properties of poly-α-L-glutamate are apparently beneficial in cellular contexts other than ribosomal modification. For example, partly amidated poly-α-L-glutamate (P-L-glx) linked to glutamine synthetase activity, is a significant component of the cell wall of virulent strains of mycobacteria (49). Interestingly, reduced levels of P-L-glx were correlated with increased antibiotic resistance and inhibition of bacterial growth (50). The role of poly-γ-glutamate (PGA) is better understood having first been described in *Bacillus anthracis* in the early 20th century. Largely present in Gram-positive bacteria, PGA performs multifunctional roles that include acting as an osmoprotectant and virulence factor (51, 52, 53). Our own observations suggest that poly-α-L-glutamate overproduction in SBW25 accounts for enhanced susceptibility to different classes of antibiotics. Increased cell wall permeability is a plausible explanation for this phenomenon.

Inevitably, the production of poly-α-L-glutamate peptides will result in the depletion of the intracellular glutamate pool. This in turn will move the solute concentration away from a position of homeostatic osmolality and result in increased glutamate transport into the cell (54, 55, 56). Increased glutamate transport through the GltP glutamate/aspartate:proton transporter will change the proton electrochemical potential of the cell to favor the uptake of antibiotics (57). Furthermore, uptake channels for carbon sources can serve as entry ports for antibiotics for which Hfq performs a central regulatory function. Thus, diverting RimK function from ribosomal modification to RimB-stimulated synthesis of poly-α-L-glutamate can account for enhanced antibiotic sensitivity by several mechanisms (30, 58, 59). Additionally, reduced glutamate levels resulting from poly-α-L-glutamate production will have consequences for nitrogen metabolism by limiting substrate availability for ammonia assimilation by glutamine synthetase. Thus, the metabolic consequences of RimK and RimB bifunctionality can be far-reaching.

**Figure 9:**
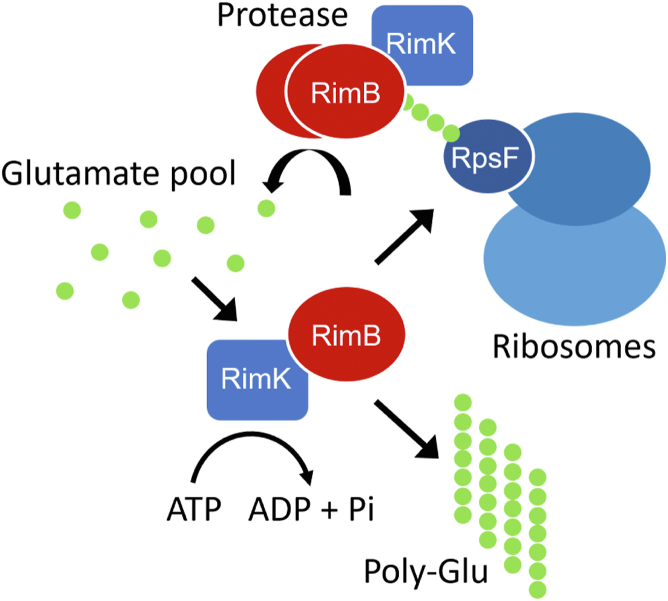
**A model for RimB regulation of ribosomal glutamylation and poly-α-L-glutamate synthesis.** RimK (*blue rectangle*) processively ligates glutamate residues to the C-terminus of ribosomal protein RpsF (*dark blue oval* positioned within the ribosome) by utilising intrinsic ATPase activity. RimB (represented by *red ovals*) acting in the dimeric state, targets the polyglutamate residue tail of RpsF for proteolysis. The equilibrium established between RimB for polyglutamate residue tails and RimK will determine the residence time on each target. Interaction of RimB with RimK results in upregulation of RimK ATPase activity and poly-α-L-glutamate synthesis. For purposes of clarity, additional components of the Rim regulatory module (namely RimA and cyclic-di-GMP) have not been included.

Taken together, our findings clearly expand the role that RimB plays as a tightly integrated component of a highly dynamic protein module. We have shown RimB to be a highly specialized bifunctional retropepsin-like protease performing a central regulatory role in determining the balance between ribosomal modification and poly-α-L-glutamate synthesis (Fig. 9).

The inclusion of the retropepsin-like *rimB* gene into the *rim* operon increases the dynamism of ribosomal modification allowing adaptive proteomic reorganization over short timescales (19, 20). Our finding that ribosomal modification is also in balance with RimB-stimulated synthesis of poly-α-L-glutamate, enhances the perception of the Rim module as a finely tuned regulator of cellular responses to the surrounding environment. Indeed, acquisition of the *rim* operon may confer an advantage in meeting the demands of specific environments.

Phylogenetic analysis revealed an interesting pattern of distribution of *rim* genes within bacterial Orders. The ‘classical’ and dominant arrangement of distinct *rimB* and *rimK* genes revealed a distribution that was not congruent with the evolution of the host organisms. This may infer that *rimB* and *rimK* are acquired through horizontal gene transfer according to a need that may reflect the environmental demands encountered by the organism; supported by the discovery that some *rim* proteins are associated with potential mobile genetic elements. Conversely, the occurrence of the *rimBK* gene was more constrained, being largely present within two bacterial Orders. We have demonstrated that the encoded RimBK protein from *D. psychrophila* possesses the same proteolytic activity as RimB; however, we did not observe glutamylation activity against ribosomal protein RpsF from SBW25 despite *rpsF* being present in the organism. Hence, RimBK may lack this activity or possess an alternative activity that has yet to be identified. This difference may feasibly reflect the different and less widespread distribution that we observe.

It is tempting to speculate that RimB may recognize other cellular targets for proteolysis. Our bioinformatic investigation revealed that *rimB*-containing genomes possess as many polyglutamate-containing genes as those genomes lacking *rimB*. Of course, in many cases, these sequences may not be accessible for RimB cleavage due to occlusion or electrostatic repulsion. Nevertheless, we have demonstrated here that RimB can cleave both terminal and internal polyglutamate protein sequences. It is noteworthy that the number of bacterial accessions possessing *rimB* or *rimBK* and genes encoding at least five terminal glutamate residues (akin to those added post-translationally to RpsF) is nearly half that of accessions lacking *rimB* or *rimBK*. Another possibility is that these sequences are targeted by related, albeit currently uncharacterized aspartic peptidases.

RimK itself may have other functions of biological relevance because for some bacteria, either no *rpsF* gene is identified in their genomes or the C-terminal sequences of ribosomal protein RpsF do not end with a glutamate residue (60). Indeed, such an example has been demonstrated in *M. jannaschii* where two RimK homologues perform roles in methanogenic coenzyme biosynthesis (61). This in turn invokes a possible role for RimB in modulating alternative modification targets. It is, therefore, a tantalizing prospect to consider that RimB, and related, RimB-like proteases may provide a regulatory function to alternative cellular processes.

## Method

### Strains and growth conditions

Strains and plasmids are listed in Table 2. Primers are listed in Table 3. Unless otherwise stated, all *P. fluorescens* strains were grown at 28 °C and *E. coli* at 37 °C in lysogenic broth (LB), solidified with 1.3% agar where appropriate. Chloramphenicol was used at 30 μg/ml, kanamycin at 50 μg/ml, and tetracycline at 12.5 μg/ml. For inducible plasmids, IPTG was used at a concentration of 1 mM unless otherwise stated. *In vivo* cleavage of the adenylate cyclase hybrid construct by RimB was assessed in *E. coli* BTH101 Δ*cya* cells (Euromedex). Prior to transformation with the desired plasmids, these cells were streaked onto LB-Strep^80^, X-Gal^40^, IPTG^0.1^ media plates and incubated at 37 °C to ensure a white coloration and therefore no Lac^+^ revertant colonies were selected. BTH101 transformant cells were subsequently grown at 28 °C in LB media.

**Table 2:** Primers used in this study

Number/Name	Sequence (5′ → 3′)	Description
1/0533NdeFor	CAGAAGCCATATGCGTCATTACGAAATC	SBW25 *PFLU0533* (*rpsF*) purification, forward primer
2/0533XhoRev 10Glu	GGTACTCGAGTTATTCTTCTTCTTCTTCTT CTTCTTCTTCTTCCTCGTCAGCGTTATCGC	SBW25 *PFLU0533* (*rpsF*) purification with ‘10Glu’ tail (Table 1), reverse primer
3/0533XhoRev 10Asp	GGTACTCGAGTTAATCATCATCATCATCATC ATCATCATCATCCTCGTCAGCGTTATCGC	SBW25 *PFLU0533* (*rpsF*) purification with ‘10Asp’ tail (Table X), reverse primer
4/0533XhoRev -1Glu-9Asp	GGTACTCGAGTTAATCATCATCATCATCATCATCATCATCTTCCTCGTCAGCGTTATCGC	SBW25 *PFLU0533* (*rpsF*) purification with ‘1Glu, 9Asp’ tail (Table 1), reverse primer
5/0533XhoRev -2Glu-8Asp	GGTACTCGAGTTAATCATCATCATCATCATCATCATCTTCTTCCTCGTCAGCGTTATCGC	SBW25 *PFLU0533* (*rpsF*) purification with ‘2Glu, 8Asp’ tail (Table 1), reverse primer
6/0533XhoRev -3Glu-7Asp	GGTACTCGAGTTAATCATCATCATCATCATCATCTTCTTCTTCCTCGTCAGCGTTATCGC	SBW25 *PFLU0533* (*rpsF*) purification with ‘3Glu, 7Asp’ tail (Table 1), reverse primer
7/0533XhoRev -Extend3	GGTACTCGAGTTATTCTTCTTCTTCTTCATCATCTTCTTCTTCCTCGTCAGCGTTATCGC	SBW25 *PFLU0533* (*rpsF*) purification with ‘Cmix4’ tail (Table 1), reverse primer
8/0533XhoRev -Extend7	GGTACTCGAGTTAATCATCATCATCATCATCTTCTTCTTCTTCCTCGTCAGCGTTATCGC	SBW25 *PFLU0533* (*rpsF*) purification with ‘Extend7’ tail (Table 1), reverse primer
9/0533XhoRev -Extend2	GGTACTCGAGTTATTCATCTTCTTCTTCATCTTCTTCTTCTTCCTCGTCAGCGTTATCGC	SBW25 *PFLU0533* (*rpsF*) purification with ‘Cmix2’ tail (Table 1), reverse primer
10/0533XhoRev -Extend1	GGTACTCGAGTTATTCTTCTTCTTCTTCATCTTCTTCTTCTTCCTCGTCAGCGTTATCGC	SBW25 *PFLU0533* (*rpsF*) purification with ‘Cmix1’ tail (Table 1), reverse primer
11/0533XhoRev -Extend11	GGTACTCGAGTTATTCTTCTTCTTCTTCTTCATCATCATCATCCTCGTCAGCGTTATCGC	SBW25 *PFLU0533* (*rpsF*) purification with ‘Extend11’ tail (Table 1), reverse primer
12/0533XhoRev -Extend4	GGTACTCGAGTTATTCTTCTTCTTCTTCTTCATCTTCTTCTTCCTCGTCAGCGTTATCGC	SBW25 *PFLU0533* (*rpsF*) purification with ‘Cmix3’ tail (Table 1), reverse primer
13/0533XhoRev -Extend8	GGTACTCGAGTTAATCATCATCATCATCTTCTTCTTCTTCTTCCTCGTCAGCGTTATCGC	SBW25 *PFLU0533* (*rpsF*) purification with ‘Extend8’ tail (Table 1), reverse primer
14/0533XhoRev -Extend9	GGTACTCGAGTTATTCTTCTTCTTCTTCTTCTTCTTCTTCATCCTCGTCAGCGTTATCGC	SBW25 *PFLU0533* (*rpsF*) purification with ‘Extend9’ tail (Table 1), reverse primer
15/0533XhoRev -Extend6	GGTACTCGAGTTATTCTTCTTCTTCTTCTTCTTCTTCATCTTCCTCGTCAGCGTTATCGC	SBW25 *PFLU0533* (*rpsF*) purification with ‘Cmix6’ tail (Table 1), reverse primer
16/0533XhoRev -Extend5	GGTACTCGAGTTATTCTTCTTCTTCTTCTTCTTCATCTTCTTCCTCGTCAGCGTTATCGC	SBW25 *PFLU0533* (*rpsF*) purification with ‘Cmix5’ tail (Table 1), reverse primer
17/0533XhoRev -Extend10	GGTACTCGAGTTATTCTTCTTCTTCTTCTTCTTCATCATCATCCTCGTCAGCGTTATCGC	SBW25 *PFLU0533* (*rpsF*) purification with ‘Extend10’ tail (Table 1), reverse primer
18/0533XhoRev -KRExtend6	GGTACTCGAGTTATTCTTCTTCTTCTTCTTCTTCTTCATCTTCCCGCTTAGCGTTATCGCTGTTG	SBW25 *PFLU0533* (*rpsF*) purification with ‘KR-Cmix6’ tail (Table 1), reverse primer
19/0533XhoRev E140K10Glu	GGTACTCGAGTTATTCTTCTTCTTCTTCTTCTTCTTCTTCTTCCTTGTCAGCGTTATCGCTG	SBW25 *PFLU0533* (*rpsF*) purification with ‘E140K-10Glu’ tail (Table 1), reverse primer
20/0533XhoRev D139K10Glu	GGTACTCGAGTTATTCTTCTTCTTCTTCTTCTTCTTCTTCTTCCTCCTTAGCGTTATCGCTG	SBW25 *PFLU0533* (*rpsF*) purification with ‘D139K-10Glu’ tail (Table 1), reverse primer
21/0533XhoRev D139A10Glu	GGTACTCGAGTTATTCTTCTTCTTCTTCTTCTTCTTCTTCTTCCTCGGCAGCGTTATCGCTG	SBW25 *PFLU0533* (*rpsF*) purification with ‘D139A-10Glu’ tail (Table 1), reverse primer
22/0533XhoRev E140D10Glu	GGTACTCGAGTTATTCTTCTTCTTCTTCTTCTTCTTCTTCTTCGTCGTCAGCGTTATCGCTG	SBW25 *PFLU0533* (*rpsF*) purification with ‘E140D-10Glu’ tail (Table 1), reverse primer
23/ColiRpsFNdeFor	CAGAAGCCATATGCGTCATTACGAAATCG	*E. coli* (K12) *b4200* (rpsF) purification, forward primer
24/EcRpsFXhoRev Mix6	GGTACTCGAGTTATTCTTCTTCTTCTTCTTCTTCTTCATCTTCCTCTTCAGAATCCCCAG	*E. coli* (K12) *b4200* (rpsF) purification with ‘Cmix6’ tail (Table 1), reverse primer
25/0840NdeFor	CAGAAGCCATATGTCGGCGACTCAAAATTACG	SBW25 *PFLU0840* (*rpsI*) purification, forward primer
26/0840XhoRev Extend5	GGTACTCGAGTTATTCTTCTTCTTCTTCTTCTTCTTCTTCTTCCTCGTCAGCGTTATCACGCTTCGAGTACTGCGGAC	SBW25 *PFLU0840* (*rpsI*) purification with ‘tail5’ tail (Table 1), reverse primer
27/0840XhoRev Extend4	GGTACTCGAGTTATTCTTCTTCTTCTTCTTCTTCTTCTTCTTCCTCGTCAGCGTTACGCTTCGAGTACTGCGGAC	SBW25 *PFLU0840* (*rpsI*) purification with ‘tail3’ tail (Table 1), reverse primer
28/0840XhoRev Extend3	GGTACTCGAGTTATTCTTCTTCTTCTTCTTCTTCTTCTTCTTCCTCGTCAGCACGCTTCGAGTACTGCGGAC	SBW25 *PFLU0840* (*rpsI*) purification with ‘tail2’ tail (Table 1), reverse primer
29/0840XhoRev Extend2	GGTACTCGAGTTATTCTTCTTCTTCTTCTTCTTCTTCTTCTTCCTCGTCACGCTTCGAGTACTGCGGAC	SBW25 *PFLU0840* (*rpsI*) purification with ‘tail1’ tail (Table 1), reverse primer
30/CyaANdeFor1	CAGAAGCCATATGCAGCAATCGCATC	*B. pertussis* CyaA purification, forward primer
31/CyaAXhoRev1	GGTACTCGAGTTATATCGATTGGCG	*B. pertussis* CyaA purification, reverse primer
32/SBW25rimBpUT18 GibHindFor1	CAATTTCACACAGGAAACAGCTATGACCATGATTACGCCATTGAAGACATTTGACCATTTGACCGT	SBW25 *PFLU0262* (*rimB*) *in vivo* expression, forward primer
33/SBW25rimBpUT18 GibHindRev1	GGTACCCGGGGATCCTCTAGAGTCGACCTGCAGGCATGCATCATGCAGCACCTGGGG	SBW25 *PFLU0262* (*rimB*) *in vivo* expression, reverse primer
34/DpRimBK-StartNdeFor	CAGAAGCCATATGTTGAGCCAAGACATTG	*D. psychrophila* RimB portion of RimBK protein purification. Forward primer.
35/DpRimBK-G163XhoRev	GGTACTCGAGTTAACCTTTTCTAGTTTCT	*D. psychrophila* RimB portion of RimBK protein purification. Reverse primer.

**Table 3:** Strains and plasmids

Strain	Description	Reference
*P. fluorescens*		
SBW25	Environmental *P. fluorescens* isolate	(63)
SBW25 *rpsF-D139K*	SBW25 with *rpsF* (*PFLU_0533*) D139K mutant allele	(19)
SBW25 Δ*rimB*	SBW25 with *rimB* (*PFLU_0262*) deleted	(19)
SBW25 Δ*rimK*	SBW25 with *rimK* (*PFLU_0261*) deleted	(19)
*E. coli*		
DH5α	*endA*1, *hsdR*17 (r_K_-m_K_+), *supE*44, *recA*1, *gyrA* (Nal^r^), *relA*1, Δ(*lacIZYA-argF*) U169, *deoR*, Φ80*dlacΔ(lacZ)*M15	(64)
BL21-(DE3) pLysS	Sm^R^, k12 *recF143 lacI*^*q*^*lacZΔ.M15, xylA,* pLysS	Novagen
Plasmids		
pETM11-*rimB*	pET*Nde*M-11 with SBW25 *rimB* as *NdeI-XhoI* fragment	(19)
pET42b(+)-*rimK*	pET42b(+) with SBW25 *rimK* as *NdeI-XhoI* fragment	(19)
pETM11-*rpsF*	pET*Nde*M-11 with SBW25 *rpsF* as *NdeI-XhoI* fragment	(19)
p25rpsF10Glu	pET*Nde*M-11 with SBW25 *rpsF* allele + 10Glu tail encoded (Table 1) as *NdeI-XhoI* fragment	This study
p25rpsF10Asp	pET*Nde*M-11 with SBW25 *rpsF* allele + 10Asp tail encoded (Table 1) as *NdeI-XhoI* fragment	This study
p25rpsF1Glu, 9Asp	pET*Nde*M-11 with SBW25 *rpsF* allele + 1Glu, 9Asp tail encoded (Table 1) as *NdeI-XhoI* fragment	This study
p25rpsF2Glu, 8Asp	pET*Nde*M-11 with SBW25 *rpsF* allele + 2Glu, 8Asp tail encoded (Table 1) as *NdeI-XhoI* fragment	This study
p25rpsF3Glu, 7Asp	pET*Nde*M-11 with SBW25 *rpsF* allele + 3Glu, 7Asp tail encoded (Table 1) as *NdeI-XhoI* fragment	This study
p25rpsF-Cmix4	pET*Nde*M-11 with SBW25 *rpsF* allele + Cmix4 tail encoded (Table 1) as *NdeI-XhoI* fragment	This study
p25rpsF-Extend7	pET*Nde*M-11 with SBW25 *rpsF* allele + Extend7 tail encoded (Table 1) as *NdeI-XhoI* fragment	This study
p25rpsF-Cmix2	pET*Nde*M-11 with SBW25 *rpsF* allele + Cmix2 tail encoded (Table 1) as *NdeI-XhoI* fragment	This study
p25rpsF-Cmix1	pET*Nde*M-11 with SBW25 *rpsF* allele + Cmix1 tail encoded (Table 1) as *NdeI-XhoI* fragment	This study
p25rpsF-Extend11	pET*Nde*M-11 with SBW25 *rpsF* allele + Extend11 tail encoded (Table 1) as *NdeI-XhoI* fragment	This study
p25rpsF-Cmix3	pET*Nde*M-11 with SBW25 *rpsF* allele + Cmix3 tail encoded (Table 1) as *NdeI-XhoI* fragment	This study
p25rpsF-Extend8	pET*Nde*M-11 with SBW25 *rpsF* allele + Extend8 tail encoded (Table 1) as *NdeI-XhoI* fragment	This study
p25rpsF-Extend9	pET*Nde*M-11 with SBW25 *rpsF* allele + Extend9 tail encoded (Table 1) as *NdeI-XhoI* fragment	This study
p25rpsF-Cmix6	pET*Nde*M-11 with SBW25 *rpsF* allele + Cmix6 tail encoded (Table 1) as *NdeI-XhoI* fragment	This study
p25rpsF-Cmix5	pET*Nde*M-11 with SBW25 *rpsF* allele + Cmix5 tail encoded (Table 1) as *NdeI-XhoI* fragment	This study
p25rpsF-Extend10	pET*Nde*M-11 with SBW25 *rpsF* allele + Extend10 tail encoded (Table 1) as *NdeI-XhoI* fragment	This study
p25rpsF-KR-Cmix6	pET*Nde*M-11 with SBW25 *rpsF* allele + KR-Cmix6 tail encoded (Table 1) as *NdeI-XhoI* fragment	This study
p25rpsF-E140K-10Glu	pET*Nde*M-11 with SBW25 *rpsF* allele + E140K-10Glu tail encoded (Table 1) as *NdeI-XhoI* fragment	This study
p25rpsF-D139K-10Glu	pET*Nde*M-11 with SBW25 *rpsF* allele + D139K-10Glu tail encoded (Table 1) as *NdeI-XhoI* fragment	This study
p25rpsF-E140D-10Glu	pET*Nde*M-11 with SBW25 *rpsF* allele + E140D-10Glu tail encoded (Table 1) as *NdeI-XhoI* fragment	This study
pEcRpsF-Cmix6	pET*Nde*M-11 with *E. coli rpsF* allele + Cmix6 tail encoded (Table 1) as *NdeI-XhoI* fragment	This study
p25rpsI-tail5	pET*Nde*M-11 with SBW25 *rpsF* allele + tail5 tail encoded (Table 1) as *NdeI-XhoI* fragment	This study
p25rpsI-tail3	pET*Nde*M-11 with SBW25 *rpsF* allele + tail3 tail encoded (Table 1) as *NdeI-XhoI* fragment	This study
p25rpsI-tail2	pET*Nde*M-11 with SBW25 *rpsF* allele + tail2 tail encoded (Table 1) as *NdeI-XhoI* fragment	This study
p25rpsI-tail1	pET*Nde*M-11 with SBW25 *rpsF* allele + tail1 tail encoded (Table 1) as *NdeI-XhoI* fragment	This study
pET42b(+)*EcRimK*	pET42b(+) with *E. coli rimK* as *NdeI-XhoI* fragment	(19)
pME-*rimK*	pME6032 with SBW25 *rimK* as *EcoRI-KpnI* fragment	(19)
pME-*Ec-rimK*	pME6032 with *E. coli rimK* as *EcoRI-KpnI* fragment	(19)
pET*Nde*M-11	Km^R^, purification vector, N-terminal His_6_-tag	(62)
pME6032	Tet^R^, P_K_, 9.8 kb pVS1 derived shuttle vector	(65)
pUCIDT-CyaA + Linker	Commercial vector encoding T18 and T25 fragments of CyaA linked by eight glutamate codons.	Integrated DNA Technologies
pCyaA+8GluLinkerM-11	T18 and T25 fragments of CyaA linked by eight glutamate codons ligated between the *NdeI* and *XhoI* sites of plasmid pET*Nde*M-11.	This study
pKNT25	pSU40-derived plasmid used with the bacterial 2-hybrid system.	Euromedex
pCyaA+8GluLinker	T18 and T25 fragments of CyaA linked by eight glutamate codons ligated between the *HindIII* and *EcoRI* sites of plasmid pKNT25.	This study
pUT18	pUC19-derived plasmid used with the bacterial 2-hybrid system.	Euromedex (40);
pUT18-SBW25	pUT18-based plasmid encoding SBW25 RimB.	This study
pDpRimBK-M11	Gene DP2392 cloned between the *NdeI* and *XhoI* sites of plasmid pET*Nde*M-11	This study
pDpsyRimB-M11	RimB-encoding portion of gene DP2392 encoding residues 1–163, cloned between the *NdeI* and *XhoI* sites of plasmid pET*Nde*M-11	This study

### Molecular microbiology procedures

Cloning was carried following standard molecular microbiology procedures. The pETM11-*rimB* and pET42b(+)-*rimK* overexpression plasmids encoding SBW25 RimB and either SBW25 or *E. coli* RimK with N-terminal and C-terminal hexa-histidine tags respectively were prepared as previously described (19). The pETM11-based *rpsF* constructs encoding SBW25 RpsF, RpsI, or *E. coli* RpsF with differing engineered tails were produced by ligating PCR fragments (amplified with primers 1–22, 25–29, and 23/24 respectively) between the *NdeI* and *XhoI* sites of plasmid pET*Nde*M-11 (62). The pME-*rimK* overexpression plasmid encoding SBW25 RimK for *in vivo* overexpression was prepared as previously described (19). Plasmid pCyaA+8GluLinkerM-11 encoding adenylate cyclase T18 and T25 fragments from *B. pertussis* linked by an eight-glutamate residue linker was produced by ligating PCR products from pUCIDT-CyaA + Linker (amplified with primers 30 & 31) between the *NdeI* and *XhoI* sites of plasmid pET*Nde*M-11. Plasmid pCyaA+8GluLinker encoding the T18 and T25 fragments of *B. pertussis* adenylate cyclase linked by an eight-glutamate residue linker was produced by digesting the commercially obtained (Integrated DNA Technologies) plasmid pUCIDT-CyaA + Linker with *HindIII/EcoRI* and inserting the resultant fragment (encoding the T18 and T25 fragments plus eight glutamate residue linker) between the *HindIII* and *EcoRI* sites of pKNT25. pUT18-SBW25rimB encoding SBW25 RimB was prepared by Gibson assembly. The PCR fragment amplified with primers 32 & 33 was ligated into *HindIII*-digested pUT18 using a Gibson assembly master mix (NEB #E2611). Gene DP2392 encoding the RimBK hybrid enzyme from *D. psychrophila* was acquired within a commercially obtained plasmid (Integrated DNA Technologies) to include an *NdeI* and *XhoI* site at the N- and C-terminus of the gene respectively. A silent mutation of a single base was made to remove an internal *NdeI* site. The gene was subsequently excised from the obtained plasmid and sub-cloned into plasmid pET*Nde*M-11 between the *NdeI* and *XhoI*. Plasmid pDpsyRimB-M11 encoding the RimB domain portion of gene DP2392 (encoding the RimBK hybrid protein from *D. psychrophila*) was produced by ligating the PCR fragment (amplified with primers 34 and 35) between the *NdeI* and *XhoI* sites of plasmid pET*Nde*M-11.

### Overexpression and purification of the rim and RpsF proteins

Cultures of *E. coli* BL21-(DE3) pLysS containing the appropriate plasmid for protein overexpression were grown in 50 ml volumes at 30 °C. These cultures (10 ml) were used to inoculate 1.0 L overexpression cultures in 2 L conical flasks. Cells were incubated at 30 °C and grown to an OD_600_ of 0.6. Protein expression was induced by the addition of IPTG to a final concentration of 1 mM. Following a further 2 h period, cells were pelleted, resuspended into 30 ml 20mM HEPES pH 6.8/200mM NaCl/2.5% glycerol, and frozen at −20 °C. For purification of the hexa-histidine-tagged proteins, cells were thawed and lysed using a cell disruptor (Avestin) and purified by Ni-NTA affinity chromatography. 1 ml HiTrap columns (Cytiva) were equilibrated with 20 mM HEPES pH 6.8/200 mM NaCl/2.5% glycerol. Following protein immobilization, non-binding proteins were washed from the column. Upon stabilization of the baseline (measured at OD_280_), the column was washed with 8% of the elution buffer (20mM HEPES pH 6.8/200 mM NaCl/2.5% glycerol/1 M Imidazole) to remove adventitiously bound contaminants. Target proteins were eluted with a 10 ml linear gradient between equilibration and elution buffers. Fractions were collected as 1 ml volumes and analyzed for purity using SDS-PAGE.

### RimB protease assays

Purified RpsF and RimK proteins at the concentrations indicated in the figure legends were incubated at room temperature for the indicated times. The reaction buffer comprised 100 mM Tris-HCl at the indicated pH. For experiments requiring longer incubation times, RpsF-Cmix6 containing a single aspartate residue (see Table 1) was routinely used. This protein digests at a slightly reduced rate relative to the all-glutamate residue equivalent protein but is more stable *in vitro*.

### Poly-**α**-L-glutamate production *in vitro*

Purified RpsF and RimK proteins at the concentrations indicated in the figure legend, were incubated at room temperature for the indicated times. The reaction buffer comprised 100 mM Tris-HCl (pH 9.0), 20 mM L-glutamate, 20 mM MgSO_4_. Reactions were initiated by the addition of ATP to a final concentration of 2.0 mM and subsequently quenched in liquid nitrogen. Samples were prepared in at least duplicate.

### Poly-**α**-L-glutamate purification

A minimum of duplicate biological replicate 50 ml cell volumes were pelleted by centrifugation, washed with 30 ml of 10 mM HEPES, pH 8.0. Cells were re-pelleted and resuspended into 1 ml of 10mM HEPES pH 8.0. Cells were lysed by mechanical disruption (Hybaid; Ribolyser), immediately placed on ice and subsequently centrifuged for 2 min at 21,000*g* in a microcentrifuge. The supernatant was then transferred to a new tube. Supernatant samples were incubated at 98 °C for 10 min and subsequently centrifuged at 4 °C, 21,000*g* for 30 min in a microcentrifuge. For every 100 μl of sample, 21.5 μl of 5 M NaCl and 200 μl EtOH was added, mixed, and placed at −80 °C for 20 min. Samples were centrifuged at 21,000*g* in a microcentrifuge for 30 min at 4 °C. The supernatant was decanted, and the poly-α-L-glutamate pellet was air-dried for approximately 3 min. Samples were resuspended into 50 to 100 μl 3% acetonitrile. Resuspended samples were subsequently vortexed for approximately 5 min and centrifuged.

### LC-MS for product analysis

High-resolution mass spectra were acquired by LC-MS on a Synapt G2-Si mass spectrometer (MS) equipped with an Acquity UPLC (Waters). Aliquots of 6 μl of the samples were injected onto an Acquity UPLC BEH C18 column, 1.7 μm, 1 × 100 mm (Waters), and eluted with a gradient of (B) acetonitrile/0.1% formic acid in (A) water/0.1% formic acid with a flow rate of 0.08 ml/min at 45 °C. The concentration of B was kept at 1% for 1 min followed by a gradient up to 60% B to 12 min. The column outlet was connected to the MS electrospray source and the eluting sample was continuously infused in the MS. Poly-α-L-glutamate products eluted between 4 and 5 min around 20% acetonitrile. The MS was controlled by the Masslynx 4.1 SCN957 software (Waters). It was calibrated with sodium formate in the mass range *m/z* 200 to 2000. Data were collected with the following parameters: resolution mode, positive ion mode, scan time 1 s, capillary voltage = 3.0 kV; cone voltage = 40 V; source temperature = 120 °C; desolvation temperature/gas flow = 350 °C/700 L/min. Leu-enkephalin peptide was used to generate a lock-mass calibration with 556.2766 m/z measured every 20 s during the run. Spectra were generated in the Masslynx software by combining spectra from the poly-α-L-glutamate elution range (4–5 min). Spectra were deconvoluted to m/z (+1) using the MaxEnt3 tool. Peaklists were exported and used to produce spectra for graphical presentation.

### Linked pyruvate kinase/lactate dehydrogenase (PK/LDH) ATPase activity assays

Simultaneous measurement of RimK ATPase activity during poly-α-L-glutamate production was achieved by the inclusion of 0.4 mM NADH, 0.8 mM phosphoenolpyruvic acid, and 0.7 μl PK/LDH (Sigma). ATPase activity was measured indirectly by monitoring NADH oxidation in a microplate spectrophotometer (BioTek Instruments) at 25 °C.

### Growth assays

Bacterial growth was measured in a microplate spectrophotometer (BioTek Instruments) using a minimum of three experimental replicates per sample. Cells were grown in rooting solution (a defined media developed for use when growing SBW25 cells in model rhizospheres) comprising 1 mM CaCl_2_.2H_2_O, 100 μM KCl, 800 μM MgSO_4_, 10 μM FeEDTA, 35 μM H_3_BO_3_, 9 μM MnCl_2_.4H_2_O, 0.8 μM ZnCl_2_, 0.5 μM NaMoO_4_.2H_2_O, 0.3 μM CuSO_4_.5H_2_O, 6 mM KNO_3_, 18.4 mM KH_2_PO_4_, 20 mM Na_2_HPO_4_ plus 0.4% w/v sodium pyruvate as carbon source. 150 μl of this media (also containing 12.5 μg/ml tetracycline and 1mM IPTG for cells containing the RimK overexpression construct) was added to each well of the microplate. Antibiotics were added to the concentrations stated in the figure legend. Growth was initiated by the addition of 5 μl of overnight cell culture (LB media, 28 °C, shaking) normalized to an OD_600_ of 0.01. Plates were covered with adhesive sealing sheets and incubated statically at 28 °C.

### **β**-galactosidase assay

LB cultures (5 ml) of each strain were started from colonies from transformation plates and grown overnight at 30 °C. 0.75 ml of each overnight culture was used to inoculate 50 ml LB with appropriate antibiotics. IPTG was added to 500 μM. Cells were grown with shaking at 28 °C. At the indicated time points, 100 μl of each culture was permeabilized with sodium dodecyl sulfate and chloroform. β-galactosidase activity was assayed using o-nitrophenyl-b-D-galactopyranoside (ONPG) as a substrate. Activities are reported in Miller units.

### Western (immuno-) blotting

To assess *in vivo* cleavage of the hybrid, adenylate cyclase construct, cell cultures were grown, and samples were prepared as follows. 5 ml overnight biological duplicate cultures of BTH101 cells containing the required plasmids were grown overnight at 28 °C with appropriate antibiotics. 0.75 ml of each overnight culture was used to inoculate 50 ml LB with appropriate antibiotics. IPTG was added to 500 μM. Cells were grown with shaking at 28 °C. At 1, 3, 5 & 7 h, 1 ml of each sample was taken and used to measure the OD_600_. This 1 ml sample was then centrifuged and resuspended into 100 μl supernatant + 100 μl 2x SDS-PAGE sample buffer. The SDS-PAGE samples were heated at 95^o^C for 5 min and then briefly sonicated to reduce viscosity. 5 μl of each sample was loaded onto a 12% pre-cast gel (Novex). Proteins were transferred onto polyvinylidene difluoride (PVDF) membranes (Millipore). Membranes were incubated overnight in a blocking solution (PBS pH 7.4, 0.01% Tween 20, 5% milk powder). Protein was subsequently detected with 1/5000 Anti-CyaA primary antibody (Santa Cruz Biotechnology, # SC-13582) and goat anti-mouse secondary antibody (Millipore # 12–349) in 1x PBS pH 7.4, 0.01% Tween 20, 1% milk powder. Bound antibody was visualized with ECL chemiluminescent detection reagent (GE Healthcare) using an ImageQuant LAS 500 imaging system (G.E. Healthcare).

### Phylogenetic analysis

A list of 5962 bacterial accessions was made that conformed to the following restrictions: they were all flagged as either "reference" or "representative" in Genbank; their genome assemblies consisted of 15 or fewer contigs; at least 500 CDSs were annotated in each genome. These accessions were searched by bidirectional BLASTp using three proteins as forward queries, DP2392 Q6AKK4 (RimB/RimK hybrid: *D. psychrophila* LSv54), PFLU_0261 (RimK: SBW25) and PFLU_0262 (RimB: SBW25). The top forward hit was used as a reverse query to search genomes from which the forward query came. Information about the reciprocity of hits was saved along with other information about the hits in the forward direction. Bespoke Perl scripts using BioPerl modules were used to carry out the BLASTp searches and parse the BLASTp output.

The results of bidirectional BLASTp searches with the three query proteins were merged into one table with separate columns for the different queries. A protein was considered to be present in an accession if the hit was reciprocal and the expected value reported by BLASTp was less than or equal to 1e-9. Accessions were separated into sets depending upon the presence or absence of the three query proteins. Accessions that had none of the three query proteins in them were removed. From the remaining 2226 accessions (Table S1), the following proteins were collected: AtpD; DhaE; GuaA; GyrB; RecA; RpoB; and RpoD and concatenated into one amino acid sequence for each accession. The concatenated proteins were aligned using MUSCLE version 5.1. After deleting columns consisting of more than 50% gaps from the alignment, a tree was made using FastTree version 2.1.11. Bespoke Perl scripts were used to make text files for annotating the tree uploaded and displayed on the iTOL website.

To analyze polyglutamate-encoding sequences, bespoke Perl scripts using BioPerl modules were used to search for proteins that contain continuous stretches of five or more glutamate residues in all annotated proteins in 12,706 reference or representative bacterial genome accessions.

## Data availability

This study includes no data deposited in external repositories.

## Supporting information

This article contains supporting Information.

## Conflicts of interest

The authors declare that they have no conflicts of interest with the contents of this article.

## Figures and Tables

**Figure S4:**