# Supplementary material for: A retropepsin-like bacterial protease regulates ribosome modification and polypeptide production
Source: J Biol Chem. 2025 Feb 18;301(3):108329. doi: 10.1016/j.jbc.2025.108329 (PMC11957791; doi:10.1016/j.jbc.2025.108329)
Supplement: Supplementary figure and table legends [file mmc1.docx]

**Figure S1. RimB proteolysis of the RpsF C-terminal tail is insensitive to common protease inhibitors. A: The rate of RimB catalysed proteolysis of RpsF is unaffected by pepstatin A or EDTA.** 12.5% SDS-PAGE gel. RpsF-10Glu was present at a concentration of 12.1 µM. RimB was present at a concentration of 0.59 µM (indicated by a single plus symbol – Lanes 3 – 9, and not visualised at this concentration) or 5.9 µM (indicated by a double plus symbol – Lanes 10 - 16). Proteolysis of RpsF is visualised by the disappearance of the band indicated in Lane 2 and by the appearance of a lower molecular weight band beneath this position. Pepstatin A and EDTA were present at a concentration of 0.3 µM, 3.0 µM or 30 µM. The increasing concentration of pepstatin A or EDTA is represented by the blue wedges. **B: The rate of RimB catalysed proteolysis of RpsF is unaffected by PMSF or 1,10-phenanthroline.** 12.5% SDS-PAGE gel. RpsF-Cmix6 was present at a concentration of 12.1 µM. RimB was present at a concentration of 0.50 µM. PMSF and 1,10-phenanthroline were present at a concentration of 1 mM. Sampling times are as shown.

**Figure S2. RimB-D31E antagonises the activity of the wild-type RimB protein.** 12% SDS-PAGE gel. SBW25 RpsF with a modified C-terminus possessing the additional residues EDEEEEEEEE was present at a concentration of 14 .5 µM. Wild-type RimB was present where indicated at a concentration of 4.8 µM. RimB-D31E was present at the following concentrations: B, 48 µM; D, 1.2 µM; E, 4.8 µM; F, 12 µM.; G, 48 µM. BSA was present where indicated at a concentration of 75 µM (not visible in the displayed region of the gel). The experiment was conducted at pH 9.0.

**Figure S3. Stability of poly-α-L-glutamate *in vitro* at varying pH.** Mass spectrometric detection of poly-α-L-glutamate polymers synthesised in the presence of 3.8 µM SBW25 RimK and 6.0 µM SBW25 RimB, subsequently purified and incubated for 1 h at room temperature in 100mM TRIS-HCl at the indicated pH prior to final purification. Numbers above a peak give the number of glutamate residues present in the polymer at that position.

**Figure S4. The RimBK hybrid protein possesses protease activity against glutamated RpsF that localises to the N-terminal RimB domain. A:** 12.5% SDS-PAGE gel. RpsF-Cmix6 was present at a concentration of 24.0 µM where indicated. RimBK was present at a concentration of 5.6 µM. Cleavage of RpsF is represented by the disappearance of the original RpsF band and the concomitant appearance of a new band at lower molecular weight. The time course of each digestion is indicated. **B:** 12.5% SDS-PAGE gel. RpsF-Cmix6 was present at a concentration of 15.0 µM. SBW25 RimB and DpRimB-Part were present at a concentration of 14 µM where indicated. Cleavage of RpsF is represented by the disappearance of the original RpsF band and the concomitant appearance of a new band at lower molecular weight. The time course of each digestion is indicated.

**Table S1. List of bacterial accession numbers and gene locations for Rim protein homologues used to create figure 8C**
